# Integrative analyses of metastatic cancer transcriptome reveal clinically distinct cellular States and ecosystems

**DOI:** 10.1038/s41598-026-36512-3

**Published:** 2026-02-05

**Authors:** Can Zhang, Si Li, Yun Yu, Meng Chi, Ziming Yuan, Kun Wang

**Affiliations:** 1 Department of Anesthesiology, The First Affiliated Hospital of Harbin Medical University, Harbin, People’s Republic of China; 2 School of Interdisciplinary Medicine and Engineering, Harbin Medical University, Harbin, 150081 Heilongjiang People’s Republic of China; 3 Department of Colorectal Cancer Surgery, The Second Affiliated Hospital of Harbin Medical University, Harbin, 150000 Heilongjiang People’s Republic of China; 4 Department of Anesthesiology, Harbin Medical University Cancer Hospital, Harbin, 150081 China

**Keywords:** Tumor microenvironment, Machine learning, Cellular states, Metastatic cancer, Clinical prognosis, Transcriptional regulation, Biomarkers, Cancer, Computational biology and bioinformatics, Oncology

## Abstract

Determining the diverse cellular states and their organization into cellular ecosystems that make up metastatic tumor is vital for elucidating the biological and prognostic diversity of cancer. However, large-scale studies profiling the clinical relevance of these cellular states and ecotypes are still lacking in metastatic cancers. In this study, we used EcoTyper, a machine learning framework, to comprehensively analyze transcriptomes from 2822 metastatic cancer patient samples covering 25 cancer types, enabling characterization of the fundamental cellular states and tumor ecosystems integral to metastatic cancer. We identified 45 distinct cellular states across 12 cell types and validated their robustness in validation cohorts. We observed that they differed in functional and prognostic associations. Survival analysis revealed that the clinically relevant cellular states, highlighting their promise as predictors of clinical outcomes. Functional enrichment analysis exhibited that the marker genes of cellular states were significantly enriched in cancer hallmark and immune-related pathways. In addition, our analysis identified five ecotypes associated with different clinical outcomes. Transcription factor enrichment analysis revealed key transcription factors (i.e. SPIB, SRF, and NR1D1) that were significantly associated with patient clinical outcomes. In conclusion, this study provided a high-resolution landscape of cellular states and ecosystems in metastatic tumors, offering new potential targets for the development of cancer treatment strategies and prognostic assessment.

**Supplementary Information:**

The online version contains supplementary material available at 10.1038/s41598-026-36512-3.

## Introduction

Tumor metastasis involves the spread of cancer cells from the primary tumor to surrounding tissues and distant organs, and is a major cause of cancer morbidity and mortality^1^. The vast majority of cancer patients die from metastatic disease rather than from the primary tumor. It is estimated that up to 90% of cancer-related deaths are due to metastatic cancer^2^. Unlike primary tumors, which can usually be cured by local therapies such as surgery and radiotherapy, metastatic cancer is a systemic disease that affects multiple organs^3^. Clinically apparent metastases remain largely incurable, but progress is being made in understanding their underlying biology and in laying the foundation for improving the long-term prognosis of patients with advanced cancer^4^. It is important to explore in depth the microenvironmental characteristics of metastatic cancer patients.

The tumor microenvironment (TME) is composed of many different cellular and non-cellular components, including cancer cells, immune cells, stromal cells, and extracellular matrix elements. The interactions between these components and tumor cells play a crucial role in tumor initiation and progression^5^. Accumulating evidence has indicated that TME can serve as a therapeutic target for cancer and can influence clinical outcomes in response to treatment^6–8^. Currently, systematic studies of the genome and transcriptome have enabled in-depth analysis of the primary TME, providing valuable insights into tumor heterogeneity^9^. However, in-depth studies of the metastatic TME are still significantly lacking.

Recent studies have introduced EcoTyper^10,11^, a tool for deciphering the cancer microenvironment from bulk data, which can better predict cancer progression, outcomes, and responses in a large number of patients. It has been successfully applied to various cancer types, such as gastric adenocarcinoma^12^, pancreatic cancer^13^, and bladder cancer^14^, among others. Six ecotypes were discovered at different stages of gastric adenocarcinoma progression and found two ecotypes associated with genomic and histopathological characteristics, and survival outcomes^12^. Previous study has identified 42 cellular states and six lung multicellular ecotypes associated with distinct radiological patterns^15^. In addition, 69 transcriptionally distinct cellular states were discovered, unveiling ten clinically relevant multicellular communities^14^. These studies demonstrated the potential of cellular states and ecotype as indicators of clinical outcomes.

Here, we collected transcriptomes from 2822 metastatic cancer patient samples and applied EcoTyper to identify the fundamental cellular states and multicellular communities that constitute metastatic tumors^11^. We identified and validated 45 transcriptionally defined cellular states across 12 cell types in the metastatic TME and investigated their associations with patient outcomes. Based on marker genes of cellular states, we comprehensively depicted the functional landscape of cellular states. In addition, we discovered 5 distinct ecotypes made up of 2 to 6 co-occurring cellular states per community, which were closely associated with patient survival. We also identified key upstream regulators of these ecotypes and demonstrated their potential as clinical biomarkers for cancer patients. In summary, these findings provided a high-resolution view of the cellular states and ecosystems that determine clinical outcomes in human metastatic tumors.

## Results

### Landscape of transcriptionally defined cellular states in metastatic cancer

**Fig. 1:**
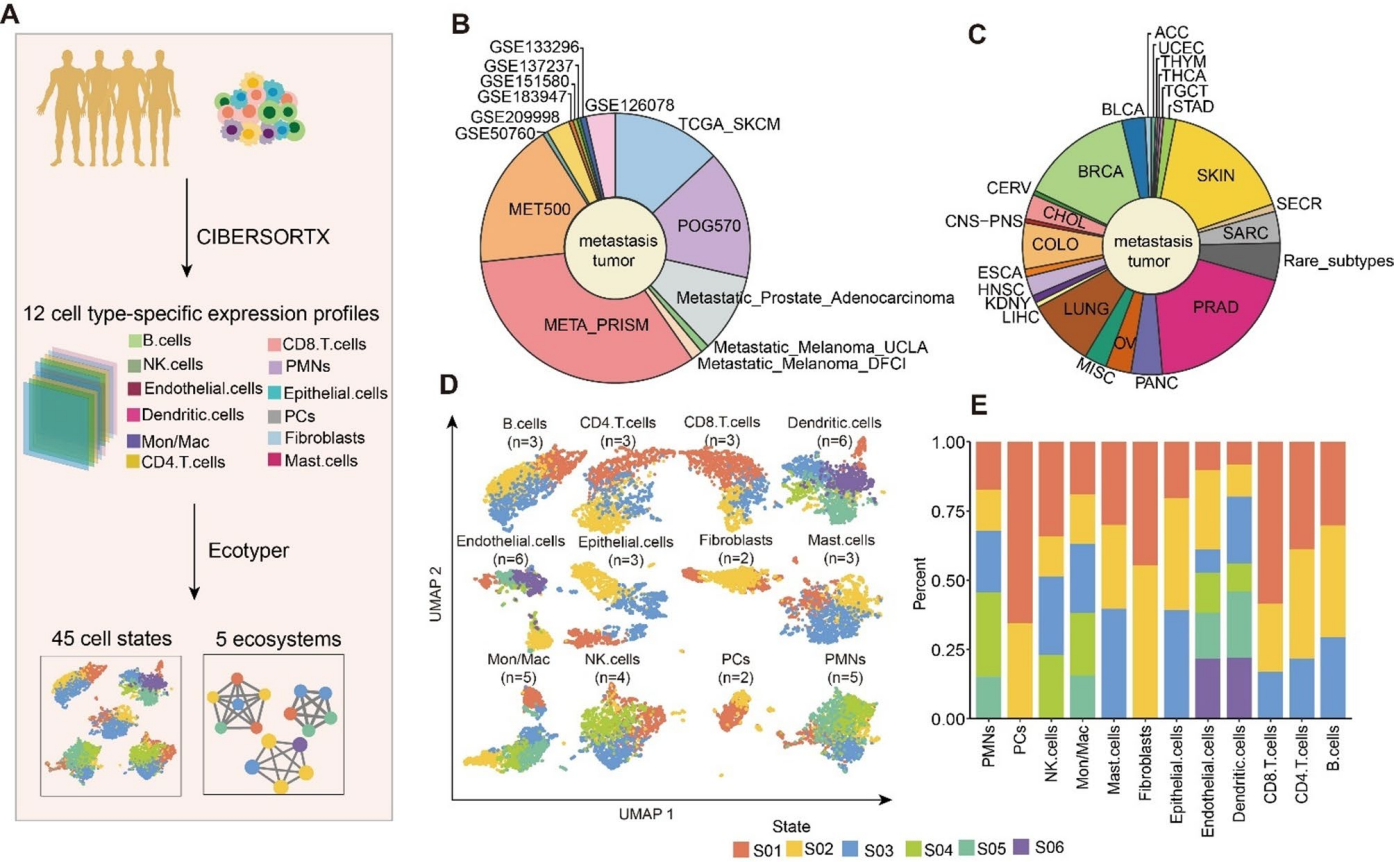
Framework for high-throughput deconstruction of cellular states and ecosystems in metastatic cancer. (**A**) Workflow for dissecting cellular states and ecosystems in metastatic cancer. (**B**) Pie chart showing the distribution of datasets in the metastatic cancer cohort. (**C**) Pie chart showing the distribution of tumor types in the metastatic cancer cohort. (**D**) UMAP plot showing cellular states of metastatic tumor patients identified by EcoTyper. (**E**) Cellular states proportion in each cell type, colored by cellular states.

We used EcoTyper to extract cell type-specific gene expression from transcriptomes for metastatic cancer patients to identify the transcriptional cellular states for each cell type. Firstly, we obtained gene expression profiles for 12 cell types using CIBERSORTx. Then, we employed Non-negative Matrix Factorization (NMF) to identify cellular states and define tumor ecosystems composed of co-occurring cellular states (Fig. 1A and Supplementary Fig. S1C-N). We integrated 2822 metastatic cancer patient samples from 14 publicly available datasets as a discovery cohort (Fig. 1B, Supplementary Table S1), covering 25 cancer types. In particular, we validated cellular states and ecotypes in independent cohorts (Supplementary Table S1). The reproducibility of the cellular states and ecotypes in validation datasets including bulk, single-cell and spatial transcriptomic datasets confirmed their stability and robustness (Supplementary Figs. S2–S8). The top three cancer types in the cohort included 544 cases of metastatic prostate cancer (19.3%), 470 cases of skin cancer (16.6%), and 401 cases of metastatic breast cancer (14.2%) (Fig. 1C).

We identified 12 cell types that make up the majority of the cellular composition found in metastatic tumors, including B cells, CD4 T cells, CD8 T cells, Dendritic cells, Endothelial cells, Epithelial cells, Fibroblasts, Mast cells, Mon/Mac, NK cells, Plasma cells (PCs), and Neutrophils (Fig. 1D). Next, we identified 45 different cellular states from 12 cell types with 2 to 6 cellular states per cell type. For example, we identified two cellular states in fibroblasts and PCs, while cellular states were most abundant in Endothelial cells and Dendritic cells (Fig. 1D). In addition, we identified three cellular states in CD4 T cells and correlated them with established functional subsets. Specifically, we compared the expression of central memory and exhaustion markers among the three states (Supplementary Fig. S9). We found that S01 exhibited high expression of exhaustion-related genes, whereas S03 showed high expression of central memory–associated genes. These findings suggested that the identified cellular states likely represented different stages of CD4 T cell functional differentiation.

Next, we compared the proportions of patients in different cellular states for each cell type (Fig. 1E) and found significant differences among the various cell types. For example, patients were predominantly in the S01 state in PCs and CD8 T cells, and predominantly in the S02 state in Fibroblasts. Studies have shown that neutrophil-cancer cell interactions played a key role in tumor metastasis^16^. For Neutrophils, we identified five cellular states. In addition, we identified two cellular states for Fibroblasts. Fibroblasts played important roles in promoting bone metastasis of prostate tumors^17^, such as increasing tumorigenic potential, promoting androgen insensitivity and favoring the metastatic process^18^. We compared the proportions of cellular states across different primary cancer types and metastatic organs and found significant differences (Supplementary Fig. S10A–L and Supplementary Fig. S11A–L). Among the three B cellular states, ACC was predominantly enriched in B cells S03, whereas UCEC was mainly enriched in B cells S02. Correspondingly, intestinal metastases primarily occurred in B cells S03, while soft tissue metastases were mainly associated with B cells S02. Among the five ecological types, ACC was predominantly enriched in E3, HNSC in E2, and SKIN in E5. These results indicated that the distribution of cellular states exhibited pronounced dual specificity with respect to organ and cancer type. Together, these results provided a landscape of cellular states in cancer metastasis patients, revealing the cellular heterogeneity of patients and offering important clues for a deeper understanding of the cellular mechanisms underlying tumor metastasis.

In addition, we compared the co-occurrence of the cellular states identified in our study between primary tumor samples and matched normal tissues in other study (Supplementary Fig. S12). Certain cellular states, such as B cells S01 and PCs S01, exhibited high consistency between primary and metastatic samples, suggesting that they may represent common features of the tumor microenvironment rather than metastasis-specific characteristics. In contrast, other states showed no such consistency, likely reflecting biological distinctions between the primary and metastatic niches. These divergent states may reveal unique features and functional specialization between the primary tumor microenvironment and metastatic ecosystems, providing new insights into tumor progression and metastasis.

### Characterization of ecosystem in metastatic cancer

**Fig. 2:**
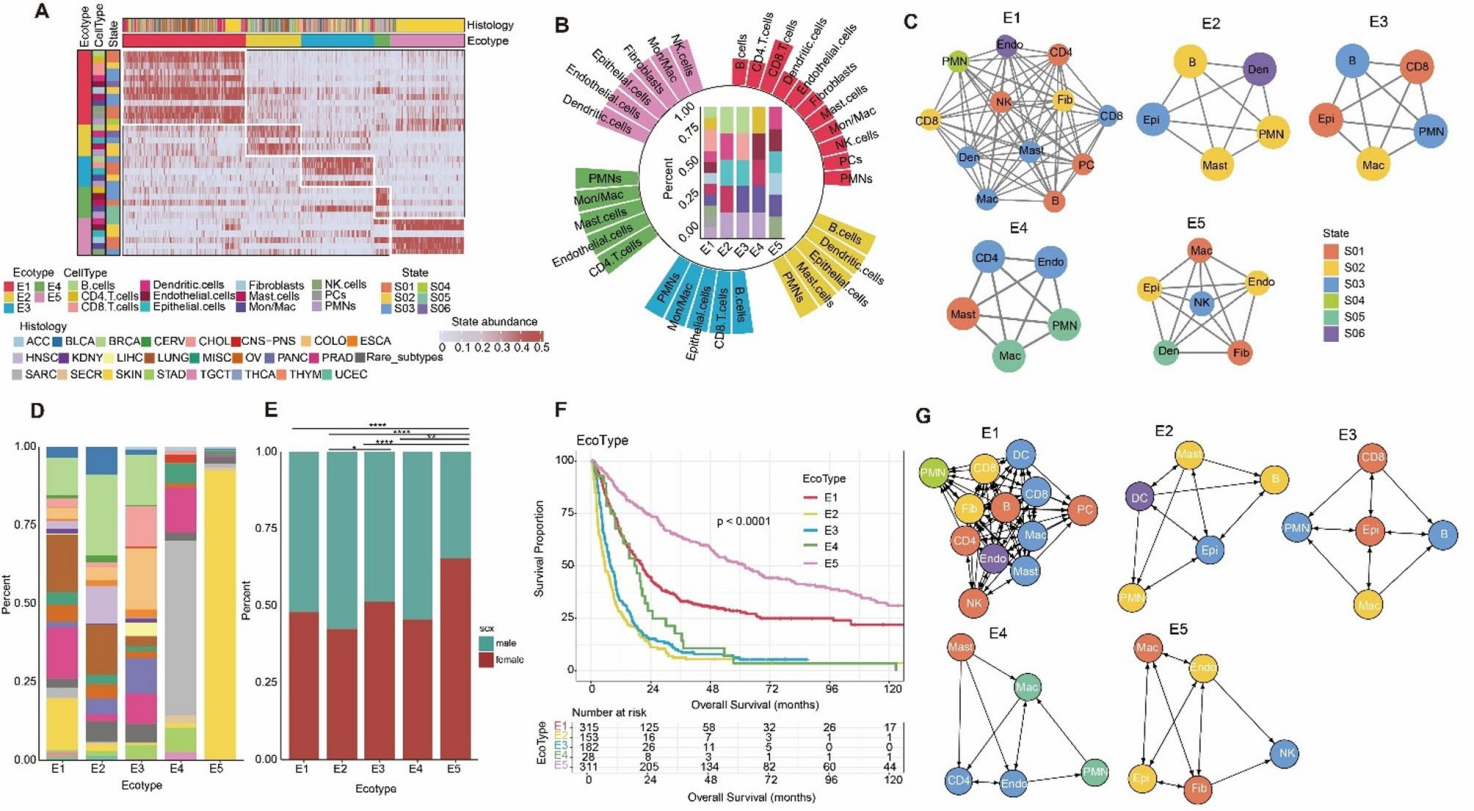
Landscape of multicellular communities in metastatic cancer. (**A**) Heatmap showing the cellular state abundances across metastatic cancer of different histologies separated into five ecotypes. (**B**) The proportion of cell types in each ecotype, colored by cell types. (**C**) Network diagrams showing five ecotypes-specific cellular states. (**D**) Cancer types proportion in each ecotype, colored by cancer types. (**E**) Sex proportion in each ecotype, colored by sex groups. *P*-values are calculated using Chi-square test, * *P* < 0.05, ** *P* < 0.01, **** *P* < 0.0001. (**F**) Kaplan–Meier analysis of patients with five ecotypes. (**G**) Network diagrams showing putative ligand-receptor interactions between cellular states within each ecotype. The arrows indicate the direction of signaling from the ligand to the receptor.

Tumors are complex and dynamic ecosystems defined by the interactions of various cell types and their microenvironment^19^. We investigated patterns of co-occurring cellular states to identify fundamental cell communities. It turns out that we discovered 5 different ecotypes (Fig. 2A) and determined the differentially expressed genes for each ecotype (Supplementary Table S2). We next calculated the proportion of cell types in each ecotype and found significant differences in cellular composition between ecotypes, with E1 and E5 being the most abundant in cell types. E1 consisted of 11 cell types and E5 consisted of 6 cell types (Fig. 2B). In addition, there were significant differences in the composition of cellular states in ecotypes, with ecotypes ranging from 5 to 12 distinct cellular states per community (Fig. 2C).

To further characterize the five ecotypes, we explored their clinical features by calculating the proportion of cancer types within each ecotype. First, we excluded the impact of batch effects on the datasets (Supplementary Fig. S1A, B). We found that prostate cancer patients were mainly distributed in E1, E3 and E4, skin cancer patients were mainly distributed in E5, and breast cancer patients were mainly distributed in E2. The proportions of cancer types varied significantly across different ecotypes (Fig. 2D and Supplementary Fig. S10M). The skin cancer samples within E5 originated from six independent datasets, thereby excluding cohort-specific bias (Supplementary Fig. S13). We further compared the proportions of ecotypes across metastatic organs and found that bone and lung metastases were predominantly enriched in E1, whereas liver metastases were mainly enriched in E3 (Supplementary Fig. S11M). Although the proportions of ecotypes varied across metastatic sites, their recurrent presence across multiple cancer types and organs supports the biological generalizability of the ecotypes. In addition, we calculated the proportion of gender in each ecotype and found that males outnumbered females in E2 and E4, whereas females outnumbered males in E5 (Fig. 2E).

Multiple studies have shown that ecotypes can predict patient outcomes^10,11^. We next evaluated the association between ecotypes and patient’s survival (Fig. 2F and Supplementary Fig. S14). We found significant differences in survival rate among patients with different ecotypes, with better prognosis observed in E5 and poorer prognosis in E2 and E3 (Fig. 2F and Supplementary Fig. S14). Next, we investigated the intercellular signaling networks within ecotypes by identifying putative ligand-receptor pairs across cellular states (Supplementary Table S3). A study on soft tissue sarcoma found that fibroblast-like cells S01 activated by TNFA/KRAS overexpressed multiple ligands in SE2, while TRIM29 + epithelial-like cells S01 and CLEC5A/SPP1 + M2-like immunosuppressive macrophages S01 were located at the core of the SE1 and SE3 signaling networks, respectively. This revealed potential cellular interactions that could shape the microenvironment of soft tissue sarcoma^20^. We also observed complex intercellular interactions between cellular states within the same ecotype (Fig. 2G). For example, Mon/Mac S03 overexpressed IL10 whose receptors IL10RA and SIRPG were overexpressed in CD4 T cells S01 and CD8 T cells S03. Similarly, epithelial S03 and S01 appeared to be at the center of the signaling networks for E2 and E3, respectively. Mon/Mac S05 and epithelial S02 appeared to be at the center of the signaling networks for E4 and E5, respectively. These results highlighted potential cellular interactions that may shape the TME of metastatic cancer.

### Functional landscape of cellular states in metastatic cancer

**Fig. 3:**
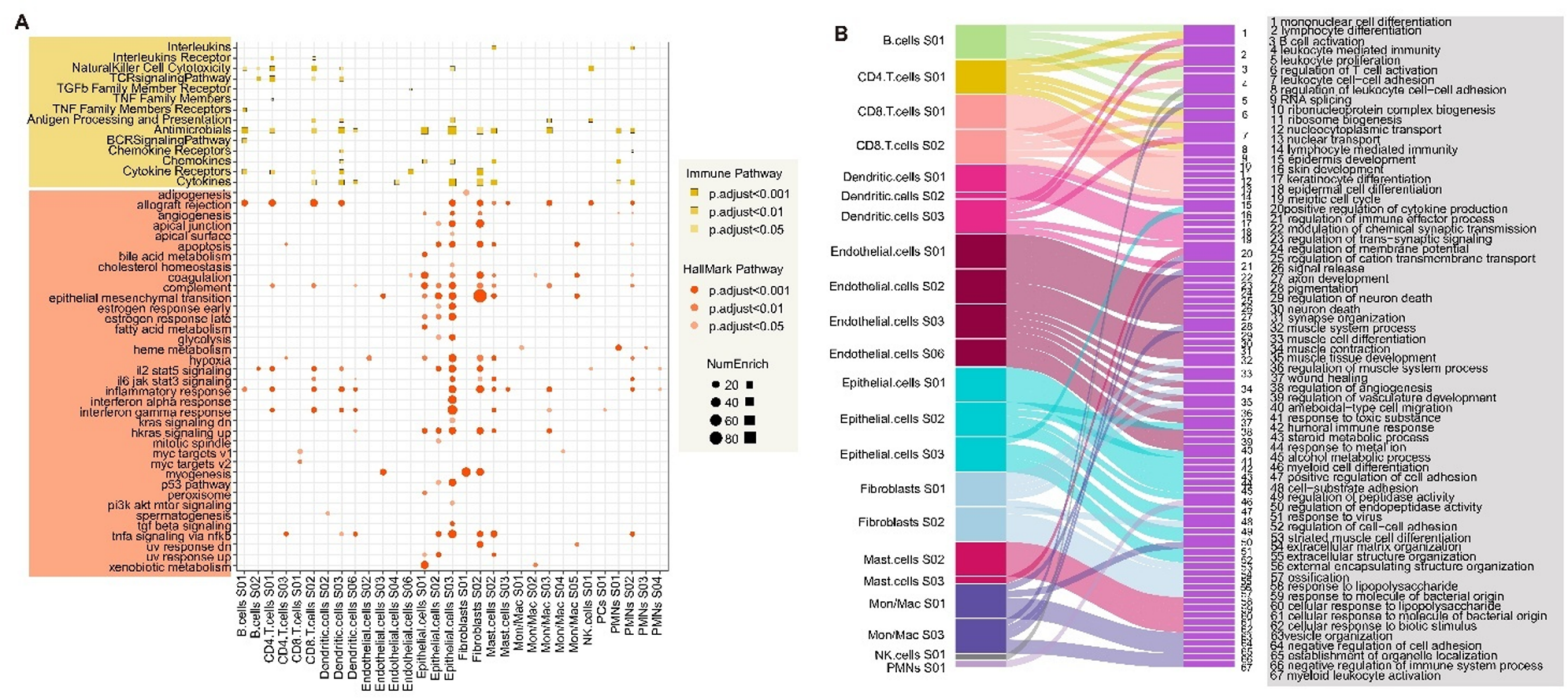
Functional landscape of cellular states in metastatic cancer. (**A**) Dot plot demonstrating the genes highly expressed of cellular states are enriched in immune-related pathways and cancer-related pathways. Circles for cancer-related pathways and squares for immune-related pathways. (**B**) Sankey diagram showing the genes highly expressed of cellular states are enriched in biological processes pathways. The left side displays the cellular states for each cell type, and the right side presents pathways.

To determine the potential biological functions of cellular states, we used hypergeometric tests to perform functional enrichment analysis of marker genes for cellular states. We found that most marker genes were enriched in immune and hallmark pathways (Fig. 3A). In particular, the marker genes for Fibroblast cells S02 and Epithelial cells S03 were significantly enriched in cancer pathways such as epithelial mesenchymal transition (EMT), kras signaling up, and hypoxia (Fig. 3A). The marker genes for CD8 T cells S02 and Dendritic cells S03 were significantly enriched in the immune pathways including cytokines, cytokine receptors, and natural killer cell cytotoxicity (Fig. 3A). Numerous studies have reported the significance of EMT in promoting metastatic phenotypes^21,22^. Moreover, accumulating evidences have indicated that hypoxia can facilitate the progression of locally advanced and metastatic cancers^23^.

Next, we analyzed the BP biological pathways enriched by marker genes for cellular states (Fig. 3B). It was found that the marker genes for B cells S01 were significantly enriched in the pathways including mononuclear cell differentiation, lymphocyte differentiation and B cell activation. CD4 cells S01 was associated with the pathways such as regulation of T cell activation, leukocyte cell-cell adhesion and lymphocyte differentiation. In addition, the marker genes of Epithelial cells S01 were enriched in response to toxic substance, steroid metabolic process and wound healing. Fibroblasts cells S02 was associated with the pathways such as extracellular matrix organization and extracellular structure (Fig. 3B). Emerging studies suggested extracellular matrix was an essential component of metastatic niches^24^. Taken together, the pathways enriched for marker genes in different cellular states depicted a functionally heterogeneous landscape.

### Prognostic atlas of cellular states in metastatic cancer

**Fig. 4:**
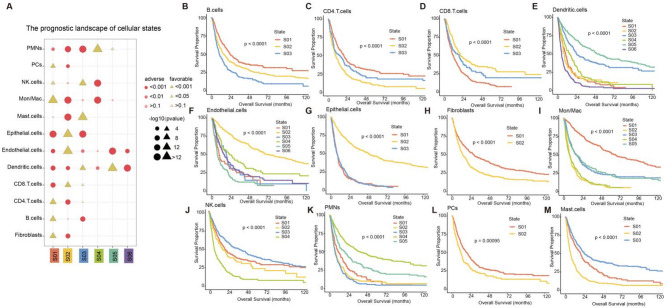
Prognostic landscape of cellular states in metastatic cancer. (**A**) Associations between cellular states and survival. Bar plots depicting the −log10 (*P*-values). Yellow represents favorable cellular states and red represents the adverse cellular states. *P*-values are calculated using log-rank tests. (**B–M**) Kaplan–Meier analysis of patients with cellular states. (**B**) for B cells. (**C**) for CD4 T cells. (**D**) for CD8 T cells. (**E**) for Dendritic cells. (**F**) for Endothelial cells. (**G**) for Epithelial cells. (**H**) for Fibroblasts. (**I**) for Mon/Mac. (**J**) for NK cells. (**K**) for PMNs. (**L**) for PCs. (**M**) for Mast cells.

Multiple studies have shown that cellular states can predict patient outcomes^10,11,25^. We depicted the prognostic atlas of 45 cellular states based on patient survival information (Fig. 4A) and found that 14 cellular states were associated with better outcomes, including Fibroblasts S01, B cells S01, CD4 T cells S01, CD8 T cells S02, Dendritic cells S03, Dendritic cells S05, Endothelial cells S02, Epithelial cells S02, Mast cells S03, Mon/Mac S01, NK cells S01, NK cells S03, PCs S01, PMNs S04. Conversely, 22 cellular states were associated with inferior outcomes, including Fibroblasts S02, B cells S03, CD4 T cells S02, CD8 T cells S01, Dendritic cells S01, Dendritic cells S02, Dendritic cells S04, Dendritic cells S06, Endothelial cells S01, Endothelial cells S03, Endothelial cells S05, Endothelial cells S06, Epithelial cells S01, Epithelial cells S03, Mast cells S02, Mon/Mac S02, Mon/Mac S04, NK cells S04, PCs S02, PMNs S01, PMNs S02 and PMNs S03 (Fig. 4A).

In addition, we explored the association between cellular states and overall survival within cell types and found significant differences in patient survival between cellular states in 12 cell types (Fig. 4B–M). For example, for immune B cells and T cells, the three B cellular states were significantly different in survival, with B cells S01 associated with better outcomes and B cells S03 associated with worse outcomes (Fig. 4A). B cells played a dual role in cancer metastasis with both pro-metastatic and anti-metastatic roles^26^, which may be related to the survival heterogeneity in different B cellular states. In addition, CD4 T cells S02 showed the worst survival while CD8 T cells S02 showed the best survival (Fig. 4C,D). Stromal cell-associated cellular states also showed significant survival heterogeneity. For example, the two fibroblast cellular states were significantly different in survival (Fig. 4H). Fibroblasts S02 was associated with worse outcomes, possibly indicating higher tumor malignancy in S02 patients. We also explored the connections between marker genes of Fibroblasts S02 and cancer progression (Supplementary Fig. S1I). For example, LAIR1 promoted the metastasis of hepatocellular carcinoma cells and was associated with poor outcomes^27^. Meanwhile, COL6A3 promoted tumor spread and metastasis in epithelial ovarian cancer^28^. It also has been shown that COL3A1 was associated with peritoneal metastasis in gastric cancer^29^. In addition, Endothelial S03 was associated with worse outcomes and showed high expression of cancer-related genes, including SYNPO2, LMOD1 and MYL9 (Supplementary Fig. S1G). In particular, it has been demonstrated that the expression of SYNPO2 was closely associated with the proliferation, migration and invasion of cancer cells in various tumors^30^. It has previously been discovered that SYNPO2 was associated with peritoneal metastasis in gastric cancer patients^30^. LMOD1 was an oncogene that regulated gastric cancer cell metastasis through the FAK-AKT/mTOR pathway^31^. Emerging studies have shown that MYL9 promoted the migration and invasion of squamous cervical cancer by enhancing aerobic glycolysis and was associated with the progression of ovarian cancer and colorectal cancer^32–34^. These findings suggested that the strong association of these genes with tumor metastasis and progression may represent an important link between particular cellular states and poor prognosis. Moreover, the six endothelial cellular states also showed heterogeneity in survival (Fig. 4F). These findings demonstrated the importance of cellular states in patient prognosis, providing new perspectives for understanding the diversity of cellular states in the TME and their impact on patient survival.

### Immune landscape for ecotypes in metastatic cancer

**Fig. 5:**
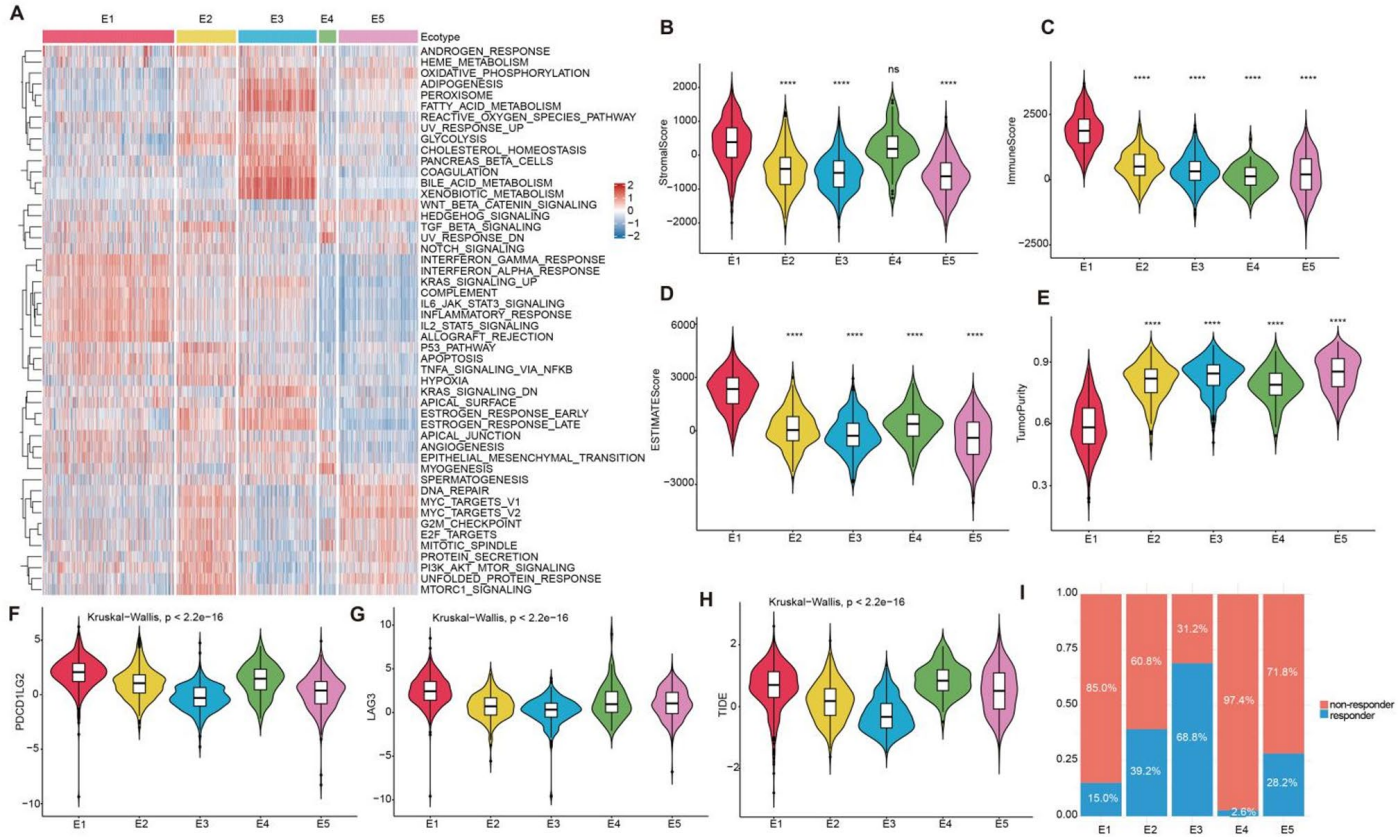
Immune landscape of five ecotypes in metastatic cancer. (**A**) Clustering heatmap displaying the scores of hallmark pathways across the five ecotypes. (**B**) Association between stromal score and five ecotypes. *P*-value is calculated using Student’s t-test. (**C**) Association between immune score and five ecotypes. *P*-value is calculated using Student’s t-test. (**D**) Association between estimate score and five ecotypes. *P*-value is calculated using Student’s t-test. (**E**) Association between tumor purity score and five ecotypes. *P*-value is calculated using Student’s t-test. (**F**) The expression of PDCD1LG2 within five ecotypes. *P*-value is calculated using Kruskal-Wallis test. (**G**) The expression of LAG3 within five ecotypes. *P*-value is calculated using Kruskal-Wallis test. (**H**) The score of TIDE within five ecotypes. *P*-value is calculated using Kruskal-Wallis test. (**I**) Differences of immunotherapy response of five ecotypes using the TIDE score.

To further characterize the ecotypes, we performed GSVA analyses to assess the differences in pathways for the five ecotypes (Fig. 5A). E1 scored higher in immune-related pathways such as interferon gamma response, interferon alpha response and inflammation-related pathways including inflammatory response, il6 jak stat3 pathway (Fig. 5A). Several lines of evidence indicated that interferon signaling played a dual role in cancer metastasis. For instance, it has been demonstrated that type I and type II interferon signaling exerted both anti-tumor and pro-metastatic effects in CRC liver metastasis, suggesting a dual mechanism of action for interferon signaling^35^. The type I interferon response in astrocytes promoted brain metastasis by enhancing the recruitment of monocytic myeloid cells^36^. Recent study has revealed that IFN-γupregulated STING in neutrophils, enhancing their cytotoxicity to eliminate tumor cells disseminated to the lungs and prevent metastasis^37^. E2 was associated with several cancer-associated pathways, such as myc targets v1, myc targets v2, e2f targets, g2m checkpoint and mtorc1 signaling, which was consistent with the association of E2 with the poor outcomes. Previous studies have demonstrated that myc targets scores were associated with tumor aggressiveness and poor prognosis in metastatic breast cancer^38^. It was also shown that RNA-binding proteins inhibited bladder cancer metastasis by downregulating the MYC pathway^39^. KRT13 promoted the growth and metastasis of breast cancer cells through the plakoglobin/c-Myc pathway^40^. Moreover, the metastatic progression of human pancreatic ductal adenocarcinoma was associated with the activation of the MYC signaling pathway in metastatic patients. These studies elucidated the association between the MYC pathway and cancer metastasis. Moreover, g2m checkpoint served as a prognostic biomarker of metastasis in estrogen receptor positive breast cancer (Fig. 5A)^41^. In particular, we observed that breast cancer patients were predominantly distributed in E2 (Fig. 2D), indicating the consistency of the results. E3 was primarily associated with metabolic pathways, such as fatty acid metabolism, bile acid metabolism and xenobiotic metabolism (Fig. 5A). These results revealed the rich functional landscape for ecotypes.

Tumor immune microenvironment played a key role in cancer development and progression^42^. To investigate the association between ecotypes and immune microenvironment, we analyzed the differences in microenvironmental components between ecotypes using the ESTIMATE algorithm^43^. E1 showed increased immune score, stromal score, and ESTIMATE score (Fig. 5B–D), indicating a higher degree of immune infiltration. In contrast, E2–E5 exhibited higher tumor purity scores compared to E1 (Fig. 5E), suggesting a higher degree of tumor malignancy.

We next compared the expression of immune checkpoints across ecotypes and found that PDCD1LG2 and LAG3 were highly expressed in E1 and E4 (Fig. 5F,G). In addition, we predicted ecotypes response to immunotherapy using the TIDE algorithm^44^, which integrated expression metrics of T cell dysfunction and T cell exclusion to assess the ability of tumor immune evasion to predict response to immune checkpoint blockade (ICB). Tumors with higher TIDE scores were more likely to trigger immune escape, which implied a lower response rate to ICB treatment. E1 and E4 exhibited higher TIDE scores (Fig. 5H,I), suggesting that patients with E1 and E4 may be insensitive to ICB therapy and are less likely to benefit from ICB treatment. In contrast, E3 had lower TIDE scores (Fig. 5H,I), indicating a greater likelihood of benefiting from ICB therapy. These findings revealed the heterogeneity of different ecotypes in the TME and their potential impact on immunotherapy response, providing a basis for personalized cancer treatment strategies.

### Regulation of ecosystems in metastatic cancer

**Fig. 6:**
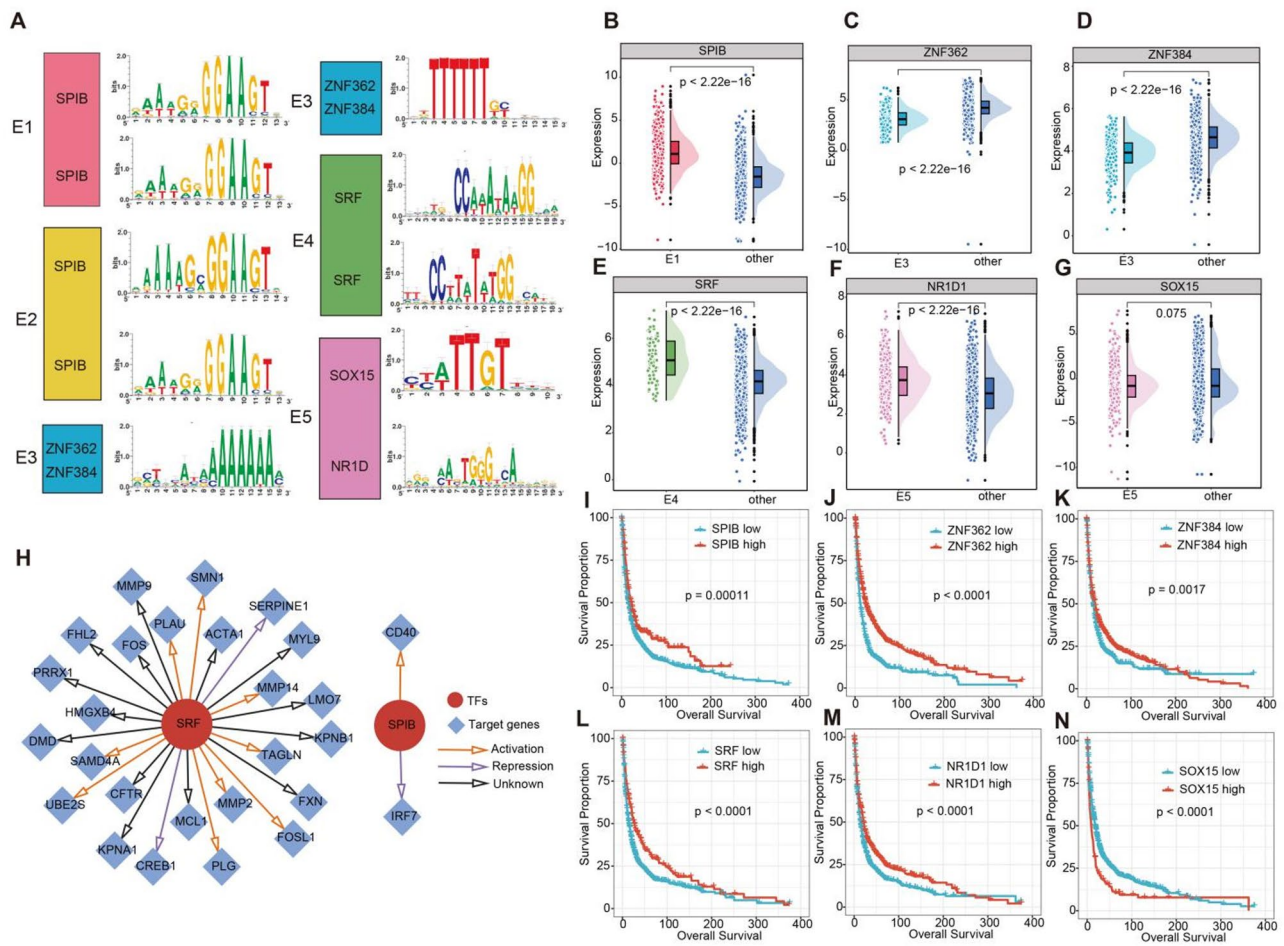
Potential regulators of ecosystems in metastatic cancer. (**A**) Representative TFs associated with five ecotypes. Top two TFs are displayed for each ecotype. (**B–G**) Boxplots displaying the expressions of representative TFs. (**B**) for SPIB. (**C**) for ZNF362. (**D**) for ZNF384. (**E**) for SRF. (**F**) for NR1D1. (**G**) for SOX15. (**H**) Network visualization showing the TF-targets interactions. (**I–N**) Kaplan–Meier curves showing overall survival stratified according to high vs. low expression. (**I**) for SPIB. (**J**) for ZNF362. (**K**) for ZNF384. (**L**) for SRF. (**M**) for NR1D1. (**N**) for SOX15.

To gain insight into the function of ecotypes, we investigated their potential upstream regulators. Transcription factors (TFs) represent potential targets for disease therapy and are associated with clinical outcomes in various cancers^45^. However, their roles in the gene regulatory networks of metastatic cancer patients still require further investigation. Based on the marker genes of ecotypes, we performed TFs enrichment analysis. We discovered key TFs for ecotypes (Fig. 6A), SPIB is a lymphocyte lineage-specific Ets transcription factor involved in mesenchymal invasion and favors metastasis in human lung cancer^46^. ZNF384 can enhance colorectal cancer (CRC) metastasis by upregulating MMP2 and may serve as a prognostic marker and regulatory factor for CRC metastasis^47^. SRF is closely associated with metastasis in various cancers, including CRC^48^, gastric cancer^49^, non-small cell lung cancer^50^ and oral squamous cell carcinoma^51^. The SOX15 gene plays a significant role in cancer growth and metastasis^52^. Overexpression of NR1D1 is associated with lung metastasis^53^.

Next, we compared the expression of TFs with high confidence in each ecotype. We observed that most TFs were highly expressed in the corresponding ecotypes (Fig. 6B–G). For example, SPIB was highly expressed in E1 (Fig. 6B), SRF was highly expressed in E4, while NR1D was highly expressed in E5 (Fig. 6E,F). In addition, we constructed a regulatory network of TFs and target genes (Fig. 6H), which contained 29 nodes and 27 interaction pairs. It was found that SRF had an activating effect on target genes such as PLAU, SMN1 and MMP2, and inhibited CREB1 and SERPINE1. SPIB played an activating role on CD40 and inhibited IRF7 (Fig. 6H). These findings provided a basis for understanding the regulatory mechanisms of TFs in distinct ecotypes.

To further investigate the potential role of TFs in patient prognosis, we analyzed the correlation between the expression levels of TFs and patient outcomes. The results showed that high expression of SPIB, ZNF362, ZNF384, SRF and NR1D1 was associated with better outcomes (Fig. 6I–M), while high expression of SOX15 was associated with worse outcomes (Fig. 6N), suggesting that SOX15 may play a pro-carcinogenic role in tumorigenesis or progression. In conclusion, these findings not only provided new perspectives for understanding the initiation and progression of the disease but also offered new potential targets for future clinical treatment and prognostic evaluation.

## Discussion

Several studies have shown that characterizing cellular states and multicellular communities in the TME is crucial for elucidating cancer biodiversity^10,11,20^. Currently, research on resolving the TME in large-scale metastatic cancer patients remains limited. In this study, we systematically analyzed transcriptomes from 2822 metastatic cancer patients using the EcoTyper machine learning framework, revealing the cellular states, multicellular communities and the complexity of their regulatory network. We identified 45 distinct cellular states and 5 ecotypes from 12 cell types, portraying a comprehensive landscape of cellular states in metastatic cancers. Furthermore, we explored the clinical significance of different ecotypes and their potential regulatory mechanisms, providing new perspectives for understanding heterogeneity in the TME.

By exploring patterns of co-occurrence among cellular states, we identified five distinct ecotypes that exhibited significant differences in both cellular composition and functional states. This finding underscores the complexity and diversity of the tumor microenvironment. A growing number of studies have shown that ecotypes are closely linked to tumorigenesis, progression, and therapeutic response. For example, previous study has identified nine ecotypes in the microenvironment of diffuse large B-cell lymphoma and reported that 89% of them were significantly associated with patient prognosis, with one ecotype capable of predicting therapeutic response^11^. Similarly, a recent study defined three ecotypes in ovarian cancer and found that a higher abundance of E3 was significantly associated with poorer prognosis. Additionally, E2 and E3 were proposed as candidate biomarkers for predicting response to therapeutic drugs^54^. In gastric adenocarcinoma, six ecotypes were identified across disease stages, two of which correlated with genomic and histopathological features as well as survival outcomes^12^. Our study also revealed a significant association between ecotypes and patient survival, with notable heterogeneity observed among different ecotypes. Furthermore, we predicted ecotype-specific responses to immunotherapy and found substantial heterogeneity in therapeutic outcomes across ecotypes. These results suggest that ecotypes could serve as potential biomarkers for predicting patient prognosis and treatment response, thereby providing a foundation for personalized therapy.

Functional enrichment analysis revealed that the marker genes of different cellular states were significantly enriched in cancer-related pathways and immune-related pathways. For example, the marker genes of CD8 T cells S02 were enriched in cytokines and cytokine receptors pathways, suggesting its anti-tumor potential. In contrast, the marker genes of fibroblasts S02 were enriched in extracellular matrix organization pathway, which may promote tumor metastasis by remodeling the TME. These functional heterogeneities were consistent with their survival heterogeneities and provided a mechanistic basis for the prognostic significance of cellular states. Prognostic mapping further revealed that 14 cellular states were associated with better survival, whereas 22 cellular states were associated with poor prognosis. These findings provided new potential targets for clinical prognostic assessment.

Through in-depth analysis of ecotypes, we discovered that E1 scored higher in immune-related pathways with greater immune infiltration, which was associated with better patient survival in E1. In contrast, E2 was enriched in multiple cancer-related pathways and was associated with the worst survival. These findings highlighted the importance of the immune microenvironment in tumor progression and provided new perspectives for immunotherapy. In addition, we identified TFs in different ecotypes and constructed a regulatory network of TFs and target genes. These TFs not only served as potential therapeutic targets, but may also be involved in regulating the dynamic change of the tumor ecosystem. In particular, the high expression of SPIB, SRF and NR1D1 was associated with better survival, while the high expression of SOX15 was associated with poor survival, which further confirmed the critical role of TFs in tumorigenesis and development.

## Conclusion

In summary, this study revealed the complex cellular states and ecosystem features in metastatic cancers through large-scale transcriptome analyses. We not only identified and validated various cellular states and ecotypes, but also deeply explored their biological functions, associations with patient prognosis, and potential regulatory network. These findings not only provided new perspectives for our understanding of disease progression, but also offered new potential targets for future clinical treatment and prognostic assessment.

## Materials and methods

### Manually collection of metastatic cancer transcriptomes

Gene expression profiles and clinical information of metastatic cancer patients were collected from GEO^55^, TCGA^56^ and published literature^57–59^. A total of 29 public datasets covering 25 cancer types were analyzed in this study. Details of the 29 datasets are listed in Supplementary Table S1. Gene expression was measured by Transcripts Per Million, where duplicates were removed based on the maximum value of gene expression in the sample.

14 datasets obtained through RNA-seq sequencing were used as the discovery cohort. Six patient-derived xenografts (PDXs) datasets were used as the validation cohort. PDX models also represented a type of metastasis^60^. Another, seven single-cell datasets and two spatial transcriptomic datasets were also used as validation cohorts. To obtain the expression profiles of the discovery cohort and validation cohort, batch correction of the datasets was performed using the “combat” function of the “sva” R package^61^. For single-cell datasets, “RunFastMNN” was used to remove batch effect.

### Cell-type-specific expression profiles

To determine the cell type-specific gene expression profiles of metastatic cancer patients, firstly we used the CIBERSORTx Fractions^62^ module to estimate cell type abundances. CIBERSORTx Fractions utilizes sets of barcode genes, termed signature matrix, to estimate cell fractions. We applied the two previously published signature matrices to dissect the main cell populations within metastatic tumors^10^. LM22 contains 22 human immune cell types^63^. The 22 subsets were aggregated into 9 major lineages: B cells, CD8 T cells, CD4 T cells, NK cells, monocytes/macrophages, plasma cells (PCs), polymorphonuclear neutrophils, dendritic cells and mast cells. TR4 contains endothelial, fibroblast, epithelial and immune populations^62^. Next, to impute cell type-specific gene expression profiles from bulk tissue transcriptomes, we used CIBERSORTx HiRes module. CIBERSORTx HiRes uses the bulk expression matrix and cell population scores of the metastatic dataset as input data. It generates cell-type-specific expression profiles for each cell population at single-sample resolution.

### Identification of cellular states in metastatic cancer

After obtaining the cell type-specific gene expression profiles, we used EcoTyper to identify clusters for each cell type^10^. EcoTyper primarily utilized non-negative matrix factorization (NMF) combined with specific heuristics to detect and quantify cellular states. Based on cell type-specific gene expression profiles, EcoTyper identified the most stable number of cellular states for each cell type by evaluating the cophenetic coefficient. The optimal number of clusters was determined based on a cophenetic coefficient closest to 0.97. To further obtain high-quality cellular states, we applied rigorous filtering criteria: cellular states with less than 10 marker genes and those identified as potential false positives according to the Adjusted False Positive Index (AFI) were omitted from subsequent analyses. EcoTyper automatically filtered out cellular states with AFI ≥ 1, thereby improving the positive predictive value of cellular state discovery. Consequently, 45 distinct cellular states, with 2 to 6 cellular states per cell type, were identified and included for further analysis.

### NMF

EcoTyper used non-negative matrix factorization (NMF) to identify transcriptionally defined cellular states from expression profiles purified by CIBERSORTx HiRes. For each cell type, NMF was applied across a range of ranks (number of cellular states), with the default range set from 2 to 20 states. For each rank, the NMF algorithm was applied multiple times using different starting seeds to ensure robustness; at least 50 runs were recommended^10^. In this study, the number of NMF runs was set to 100 to ensure reproducibility and stability of the results.

### Discovery of ecotypes

We employed EcoTyper to detect conserved cellular communities, referred to as ecotypes, characterized by co-occurring cellular states^10^. Initially, a binary matrix *A* is created, where cellular states correspond to rows and samples represent columns. If cellular state *i* is assigned to sample *j*, *A*_*ij*_ is set to 1; otherwise, *A*_*ij*_ is set to 0. We used the jaccard index to quantify the degree of overlap between cellular states in *A*, generating a 45*45 jaccard matrix based on the number of cellular states. The hypergeometric test was used to assess statistical significance.

Next, we used the function “hclust” in the R package “stats” to perform unsupervised hierarchical clustering for jaccard index matrix. Finally, by maximizing the silhouette width, we determined the optimal number of clusters and clusters with ≤ 3 cellular states were removed from further analysis. Using this method, we identified 5 distinct ecotypes.

To estimate each ecotype abundance, the abundance of cellular states was averaged within each of the three ecotypes. In each sample, the resulting values were normalized to sum to 1 across all ecotypes. To assign samples to ecotypes, we used a two-sided t-test with unequal variances to assess the difference between the estimated abundance of cellular states in each ecotype relative to the estimated abundance of all cellular states across other ecotypes. Using the Benjamini-Hochberg method, the obtained *P*-values were corrected for multiple hypothesis testing. Provided that the q-value was ≤ 0.25 and the sample was allocated to at least one cellular state within the ecotype, the sample was categorized to the ecotype with the highest abundance. Network diagrams depicting each ecotype were generated in Cytoscape^64^. Edge thickness represents the Jaccard index across samples.

### Intercellular signaling network

To investigate the signaling networks within ecotypes, we performed enrichment analysis of predicted ligand–receptor pairs using CIBERSORTx-deconvoluted cell-type-specific gene expression profiles derived from metastatic samples in the discovery cohort^65^. Samples were categorized based on the most abundant cellular state. We evaluated whether each ligand and receptor were overexpressed in samples corresponding to each cellular state compared to all other samples based on one-sided Mann–Whitney U-tests. *P*-values were adjusted for multiple hypothesis testing, and significantly overexpressed ligand–receptor pairs across different cellular states were identified.

### Single-cell sequencing of metastatic cancer

We gathered metastatic scRNA-seq transcriptome data from the Gene Expression Omnibus (GEO, http://www.ncbi.nlm.nih.gov/geo/). Quality control has been performed for each dataset in the original study. The unique molecular identifier (UMI) counts were normalized based on “NormalizedData”. Next, the data were scaled based on “ScaleData”. The function of “FindVariableFeatures” was used to identify highly variable genes. Downstream analysis was performed based on the top 3000 highly variable genes.

The “RunPCA” function was applied to perform dimensional reduction and the top 40 principal components (PCs) were selected for downstream analysis. Next, cells were divided into diverse clusters utilizing the “FindNeighbors” function and “FindCluster” function. “RunFastMNN” was used to remove batch effect. Finally, Uniform Manifold Approximation and Projection (UMAP) was used for visualization.

### Recovery of cellular States from scRNA-seq

The recovery of cellular states from scRNA-seq data was performed by mapping annotated single-cell transcriptomes to EcoTyper states. To assess the statistical significance of cell-state recovery, permutation testing was employed to generate z scores, which measure the statistical confidence^10^. Only the cellular states identified within each scRNA-seq dataset were considered, and the resulting z scores were aggregated into a meta z score using Stouffer’s method.

### Recovery of EcoTyper cellular states and ecotypes from spatial transcriptomics data

We applied EcoTyper to quantitate 45 cellular states and five ecotypes in spatial transcriptomics (ST) arrays of pancreatic cancer (1 section), colorectal cancer (1 section). Cellular state and ecotype abundance within each spatially barcoded spot were estimated by imputing the fractional abundance of each cellular state in the Visium array using EcoTyper. The most abundant cellular state for each cell type within a spot was assigned a value of 1, while the remaining cellular states were assigned a value of 0. Each cellular state was then normalized by multiplying it by the parent cell type fraction obtained from CIBERSORTx. Ecotype abundance for each spot was computed by averaging the relative fractions of each cellular state within the corresponding ecotype. Ecotype abundances were scaled so that the 99th percentile value across all three ecotypes was set to 1.

### Comparative analysis of cellular states between metastatic and other study

To assess the similarity of cellular states between the metastatic cohort and other study^10^, we performed systematic gene co-occurrence analysis. Marker genes for each cellular state were extracted from both cohorts across major cell types including immune, stromal, and epithelial cells. For each pairwise comparison between other study and metastatic cellular states, we calculated the overlap of marker genes and derived the odds ratio (OR) with one-sided Fisher’s exact test.

### UMAP visualization of cellular states

The “umap” R package^66^ was used to visualize the cellular states in 12 cell types. We analyzed the gene expression profiles imputed by CIBERSORTx in the discovery cohort and we used the euclidean distance for this analysis. Then, the resulting distance matrix was used to create Uniform Manifold Approximation and Projection (UMAP) embedding for each cell type.

### Functional enrichment analysis of cellular states

We performed functional enrichment analysis of marker genes for cellular states from EcoTyper. The “org.Hs.eg.db” and “clusterProfiler”^67^ R packages were used to perform functional enrichment analysis. The function of “enrichGO” in “clusterProfiler” was used to perform Gene Ontology (GO) enrichment analysis, we set the parameters “ont” = “BP”, “maxGSSize” = 15 and “maxGSSize” = 500.

To investigate the association between each cellular state and cancer hallmark pathways and immune-related pathways, we obtained the immune-related pathways from ImmPort^68,69^ and cancer hallmark pathways from MSigDB^70^. In total, 50 cancer hallmark pathways and 17 immune pathways were downloaded for analysis. Hypergeometric test was used to calculate the enrichment *P*-value to test whether the marker genes of cellular states were significantly enriched in these pathways. “ggplot2” R package was used to visualize the enriched pathways.

### Identification of transcriptional factors for ecotypes

Wilcoxon’s rank sum test was used to obtain the highly expressed genes within ecotypes. Genes with p.adjust < 0.05 were selected for Rcistarget analysis to identify transcription factors (TFs) associated with ecotypes^71^. The gene-motif sequencing database used by RcisTarget was “hg19-tss-center-10 kb-7species.mc9n.feathe”. The main functions “calcAUC”, “addMotifAnnotation” and “addSignificantGenes” were used with default parameter. The top two motifs were visualized for each ecotype.

To construct the TFs-target network, we downloaded the TFs-target relationship from TRRUST v2, which contained the regulatory relationships of 8,427 TF targets of 795 human TFs, and also provided information on the mode of regulation (activation or repression)^72^. we visualized the TFs-target network based on Cytoscape^64^.

### Survival analysis of cellular states

To assess the association between cellular states and patient outcomes, survival analysis for cellular states was performed based on the “survfit” function from the “survival” R package. The survival curves were plotted using the “ggsurvplot”function. Cellular states with *P*-value < 0.05 were considered to be significantly associated with patient survival.

### Characterization of immune landscape for ecotypes

To identify immune characteristics of five ecotypes, we used the ESTIMATE algorithm to calculate the stromal score, immune score, estimate score and tumor purity^43^. We used the TIDE score to predict ecotype response to immunotherapy, with a lower score indicating a better response to immunotherapy.

The TIDE score was calculated using the Tumor Immune Dysfunction and Exclusion database (http://tide.dfci.harvard.edu/login/).

### Statistical analysis

Continuous data were compared using appropriate statistical tests, including Wilcoxon rank-sum test, or Kruskal–Wallis test. Categorical data were analysed using the Fisher’s test. The log-rank test was employed to determine statistical significance in survival analysis. A *P*-value < 0.05 was considered indicative of a significant difference for all statistical tests. R software was utilized for all statistical analyses.

## Supplementary Information

Below is the link to the electronic supplementary material.

Supplementary Material 1

Supplementary Material 2

## Data Availability

Public gene expression profiles used in this work can be acquired from Gene Expression Omnibus (GEO, http://www.ncbi.nlm.nih.gov/geo/) and TCGA Research Network portal (https://portal.gdc.cancer.gov/). All datasets used in this study are provided in Supplementary Table S1.
