# Supplementary material for: Integrative analyses of metastatic cancer transcriptome reveal clinically distinct cellular States and ecosystems
Source: Sci Rep. 2026 Feb 5;16:7343. doi: 10.1038/s41598-026-36512-3 (PMC12923827; doi:10.1038/s41598-026-36512-3)
Supplement: Supplementary file 2 — Supplementary Material 2 [file 41598_2026_36512_MOESM2_ESM.docx]

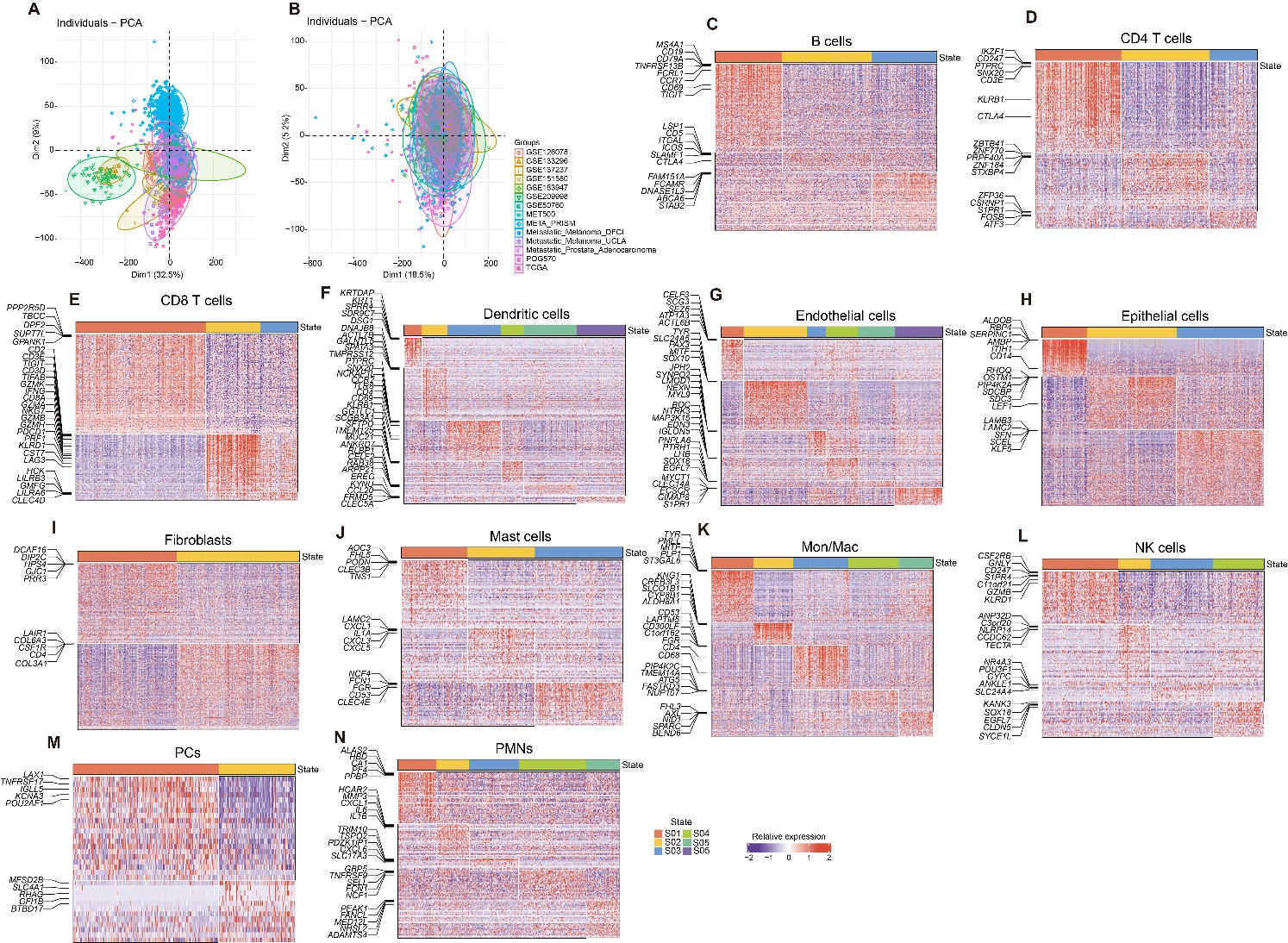


**Supplementary Fig. S1. The landscape of cellular states in discovery cohort. (A)** PCA plot showing the different datasets in the discovery cohort before batch effect removal. **(B)** PCA plot showing the different datasets in the discovery cohort after batch effect removal. (**C-N)** Heat map showing cellular states identified from metastatic cancer bulk transcriptomes in the discovery cohort. (**C)** for B cells. **(D)** for CD4 T cells. **(E)** for CD8 T cells. **(F)** for Dendritic cells. **(G)** for Endothelial cells. **(H)** for Epithelial cells. **(I)** for Fibroblasts. **(J)** for Mast cells. **(K)** for Mon/Mac. **(L)** for NK cells. **(M)** for PCs. **(N)** for PMNs.


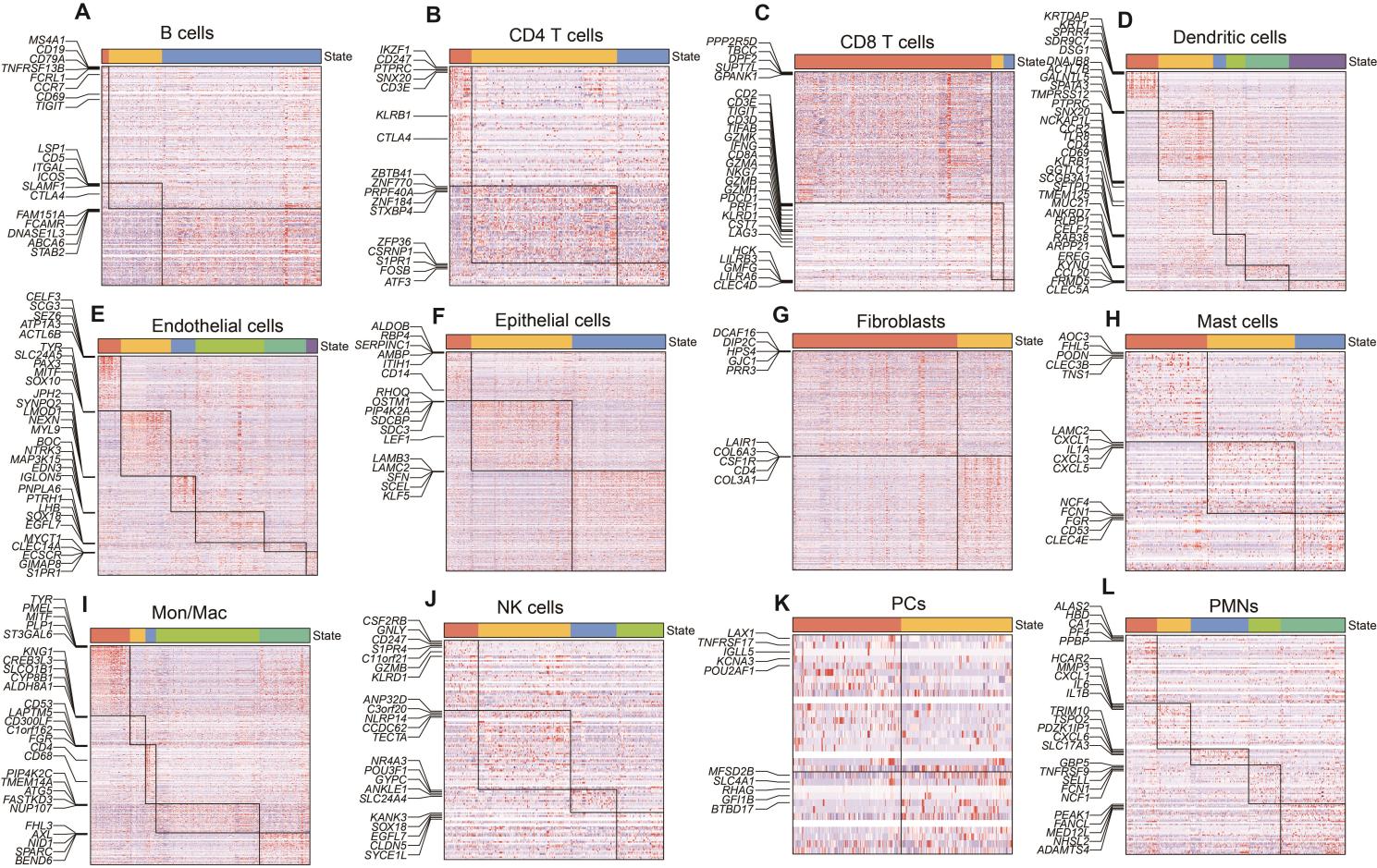


**Supplementary Fig. S2. The landscape of cellular states in validation cohort.** (**A-L)** Heat map showing cellular states identified from metastatic cancer bulk transcriptomes in the discovery cohort. (**A)** for B cells. **(B)** for CD4 T cells. **(C)** for CD8 T cells. **(D)** for Dendritic cells. **(E)** for Endothelial cells. **(F)** for Epithelial cells. **(G)** for Fibroblasts. **(H)** for Mast cells. **(I)** for Mon/Mac. **(J)** for NK cells. **(K)** for PCs. **(L)** for PMNs.


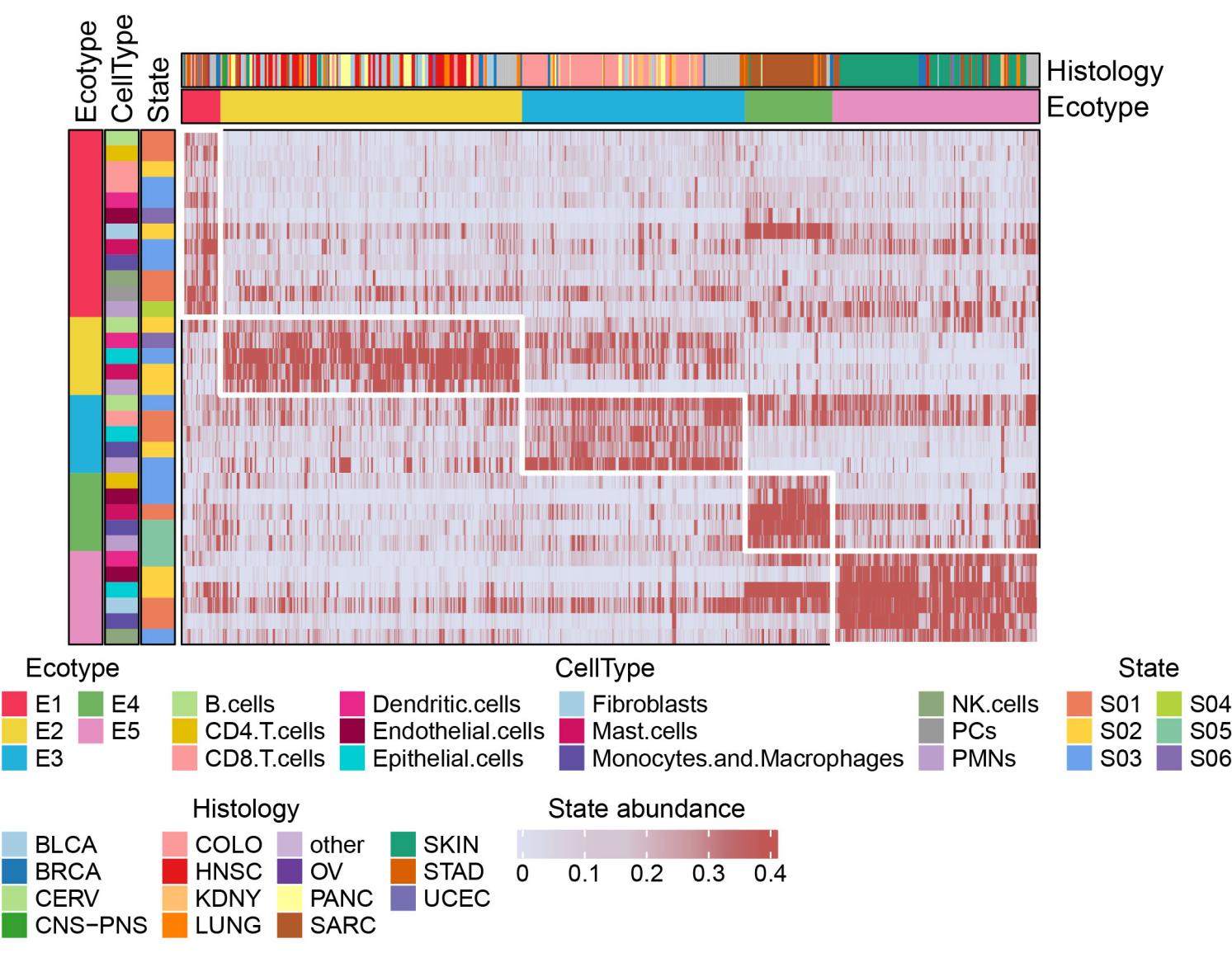


**Supplementary Fig. S3. The landscape of ecotypes in validation cohort.** Heatmap showing the cellular state abundances across metastatic cancer of different histologies separated into five ecotypes in validation cohort.


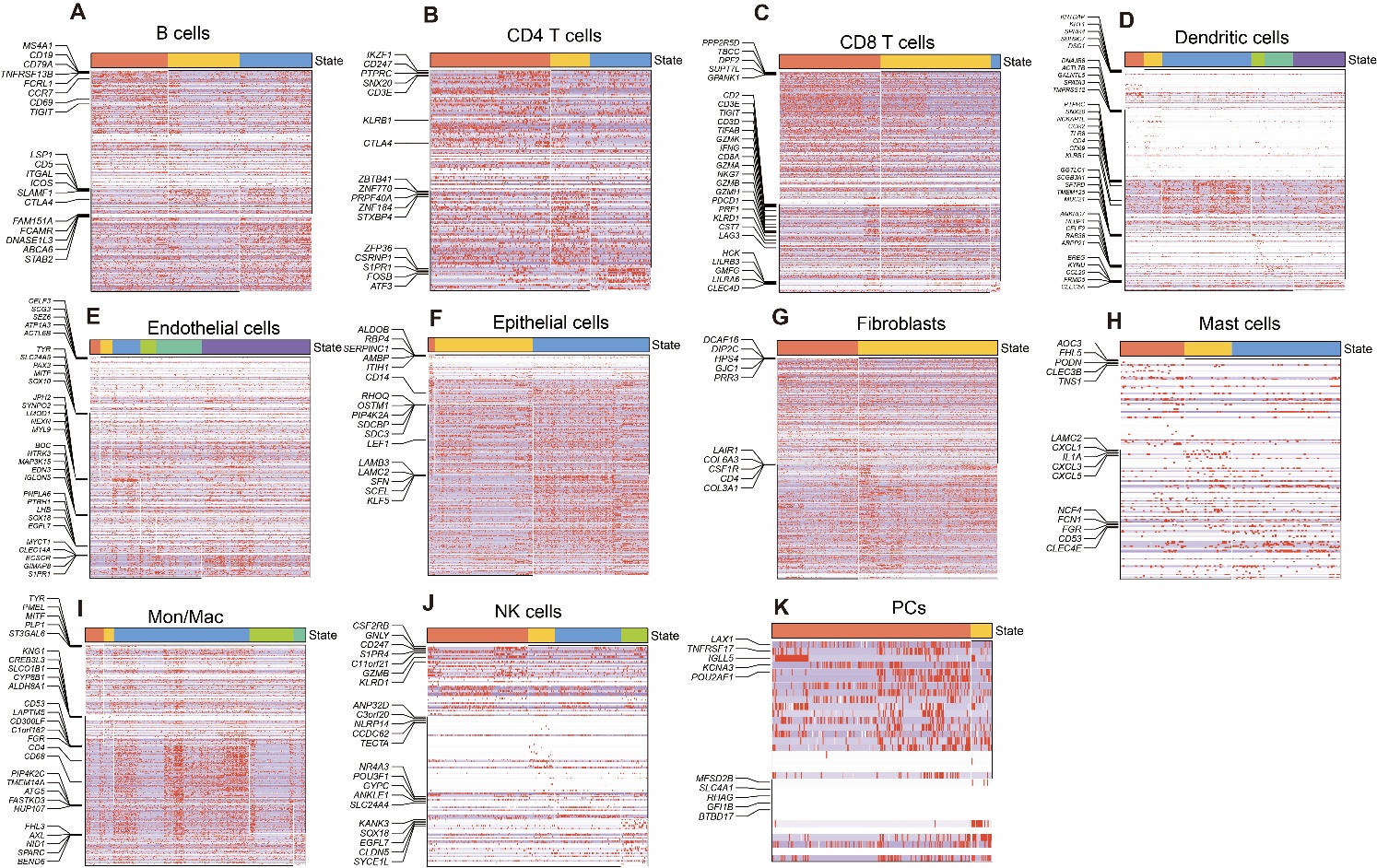


**Supplementary Fig. S4.** **The landscape of cellular states in validation single cell cohort**. **(A-K)** Heat map showing cellular states identified from metastatic cancer bulk transcriptomes in the discovery cohort. **(A)** for B cells. **(B)** for CD4 T cells. **(C)** for CD8 T cells. **(D)** for Dendritic cells. **(E)** for Endothelial cells. **(F)** for Epithelial cells. **(G)** for Fibroblasts. **(H)** for Mast cells. **(I)** for Mon/Mac. **(J)** for NK cells. **(K)** for PCs.


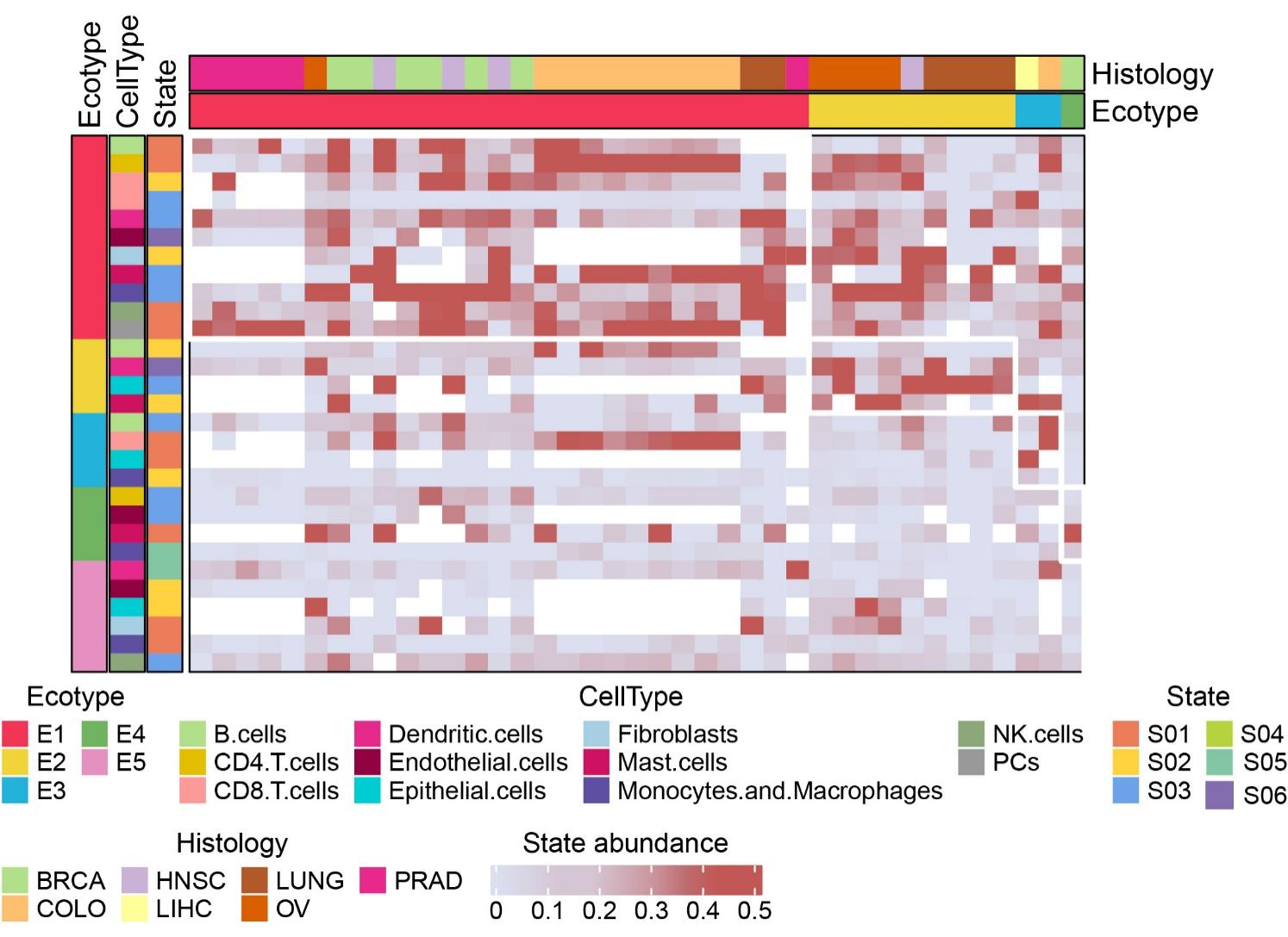


**Supplementary Fig. S5. The landscape of ecotypes in validation single cell cohort.** Heatmap showing the cellular state abundances across metastatic cancer of different histologies separated into five ecotypes in validation single cohort.


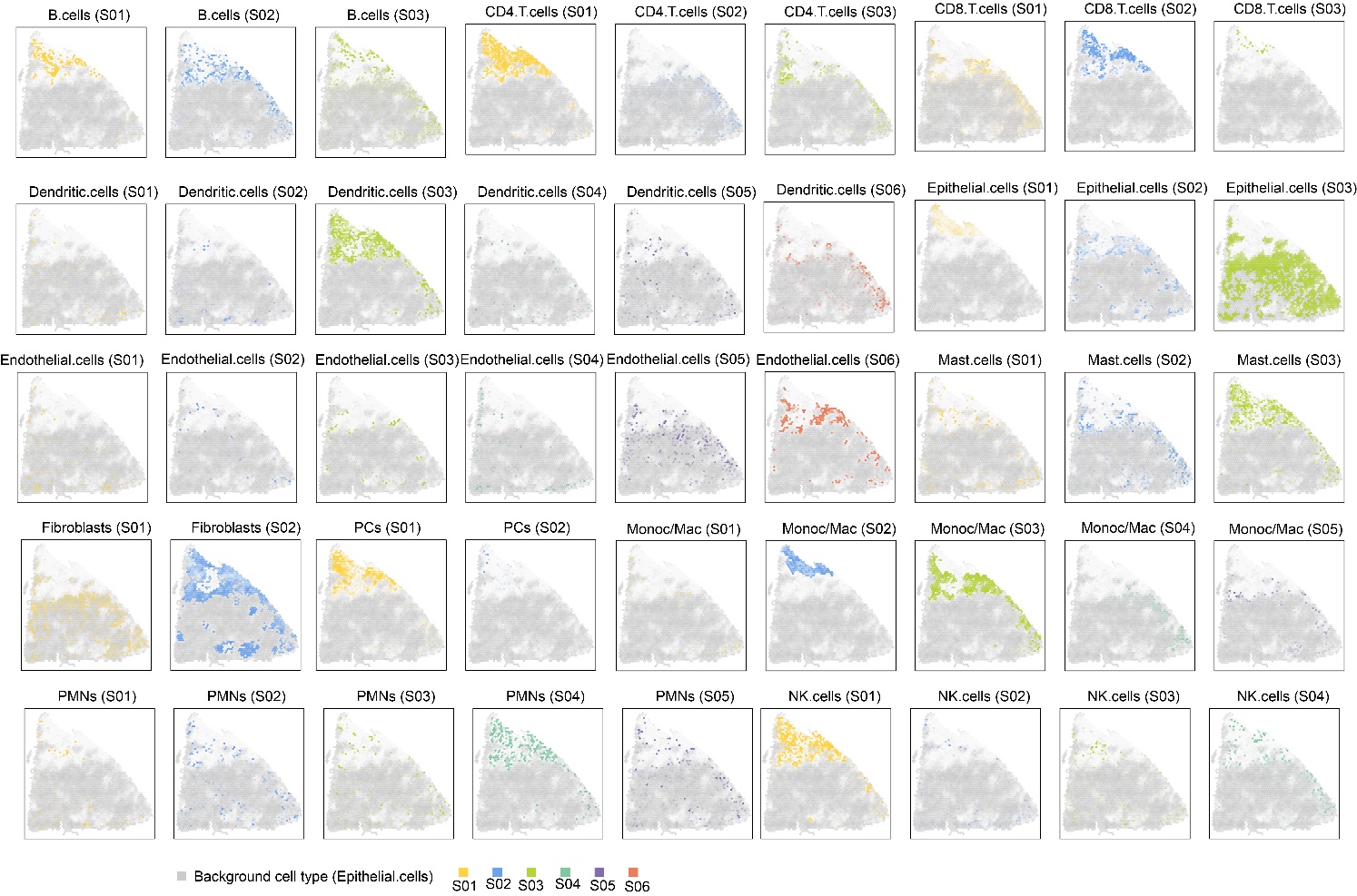


**Supplementary Fig. S6. The landscape of cellular states in validation spatial transcriptomic cohort.** Distribution of cellular states in pancreatic tumor section profiled by spatial transcriptomics.


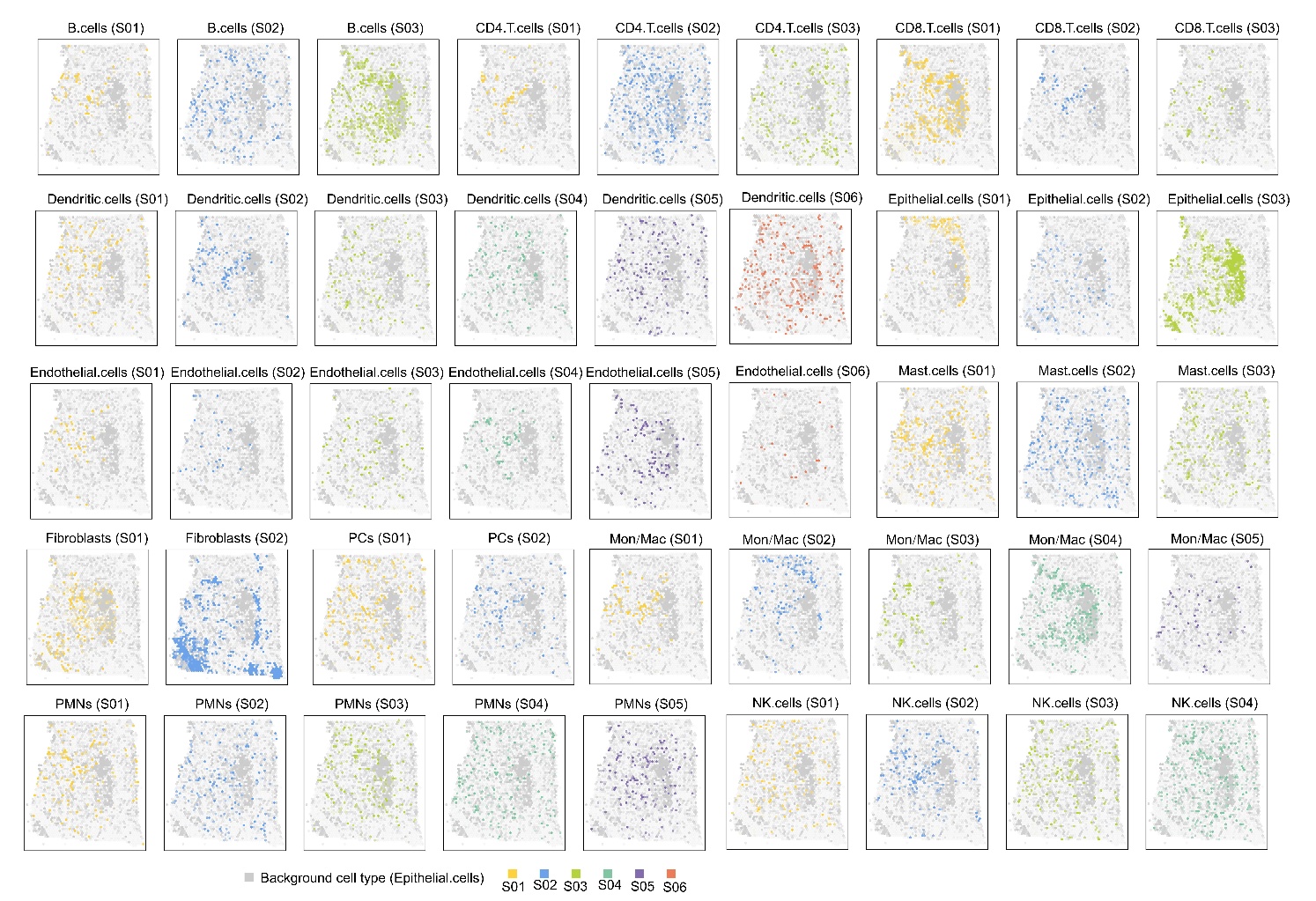


**Supplementary Fig. S7. The landscape of cellular states in validation spatial transcriptomic cohort.** Distribution of cellular states in colorectal cancer sections profiled by spatial transcriptomics.


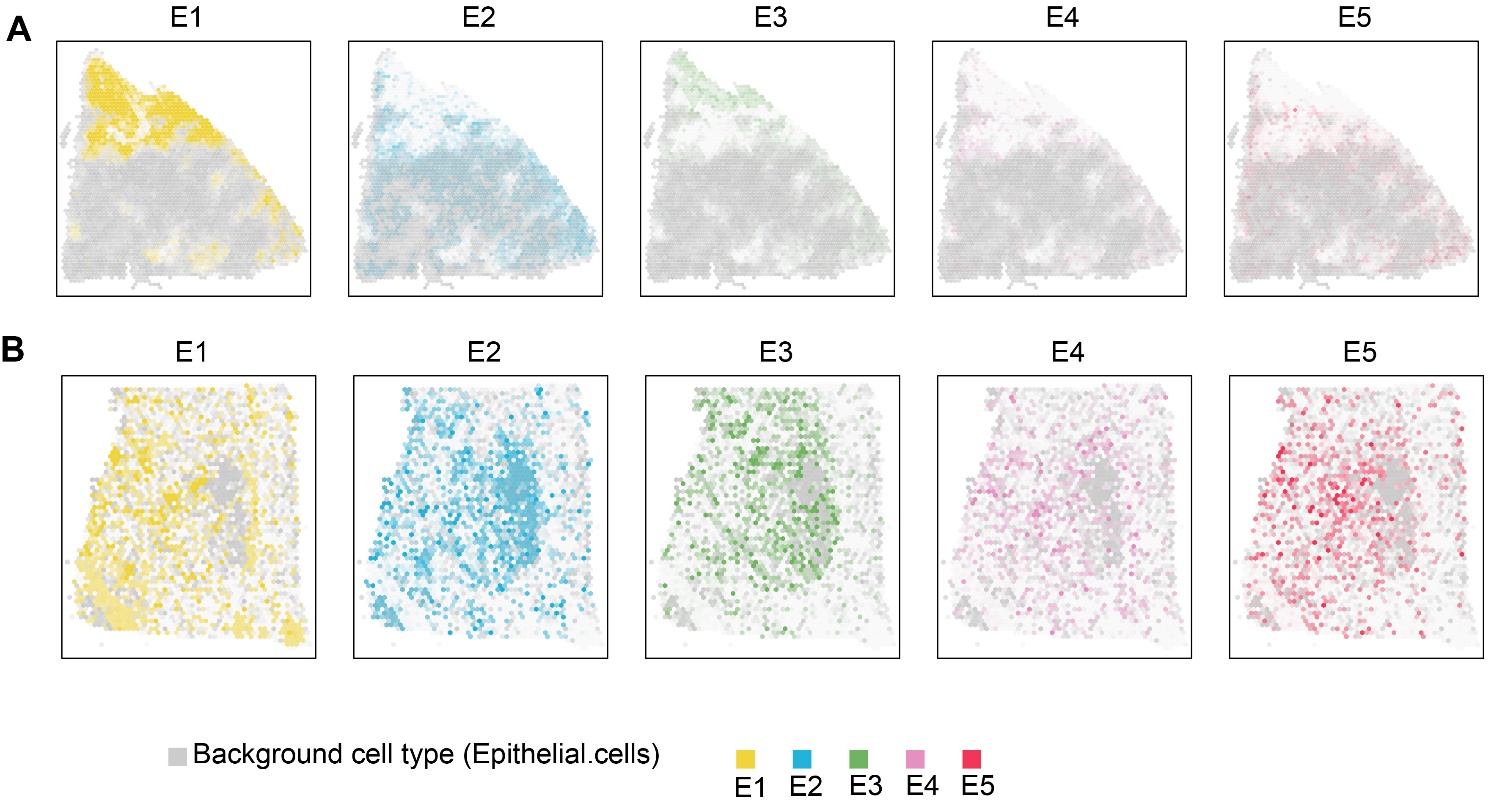


**Supplementary Fig. S8. The landscape of ecotypes in validation spatial transcriptomic cohort.** Distribution of ecotypes in colorectal cancer sections profiled by spatial transcriptomics.


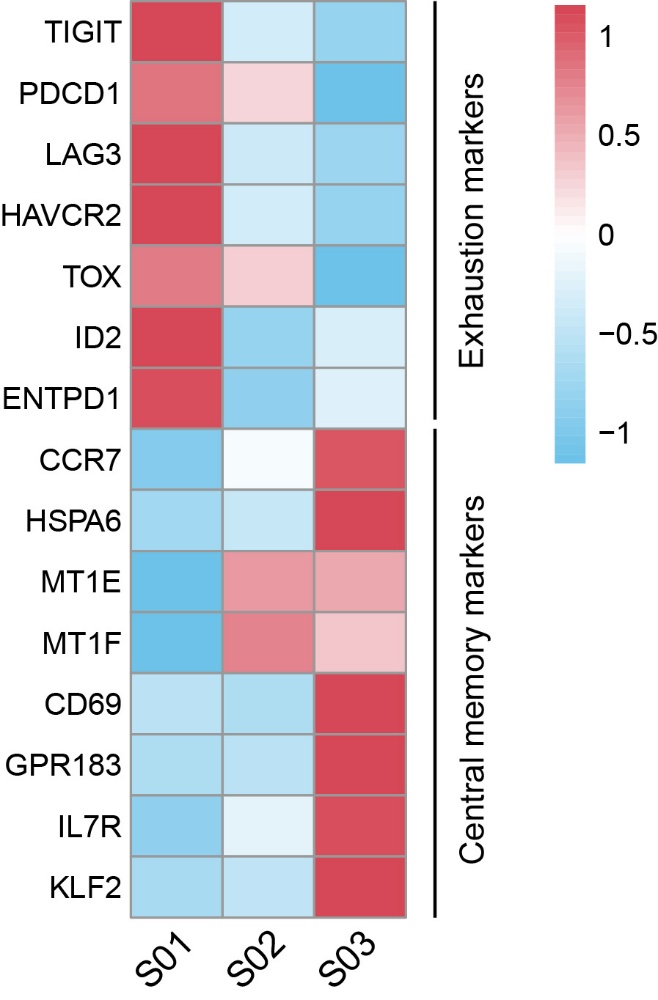


**Supplementary Fig. S9.** The expression of central memory and exhaustion markers among the three CD4 T cellular states.
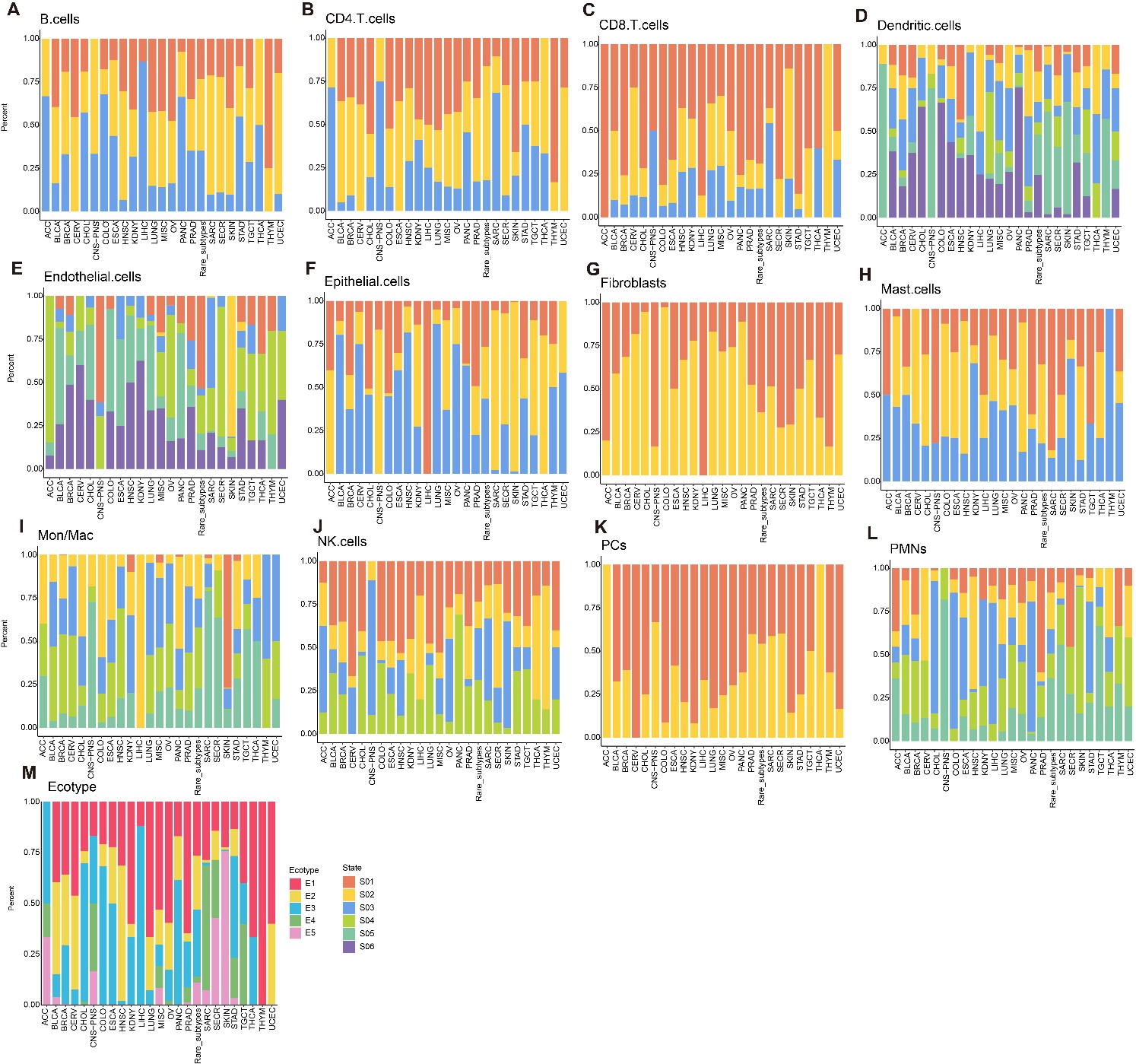


**Supplementary Fig. S10. The landscape of cellular states and ecotypes across different cancer types.** Proportions of cellular states and ecotypes across different cancer types. **(A)** for B. cells. **(B)** for CD4.T.cells. **(C)** for CD8.T.cells. **(D)** for Dendritic.cells. **(E)** for Endothelial cells. **(F)** for Epithelial.cells. **(G)** for Fibroblasts. **(H)** for Mast.cells. **(I)** for Mon/Mac. (J) for NK.cells. **(K)** for PCs. **(L)** for PMNs. **(M)** for Ecotype.


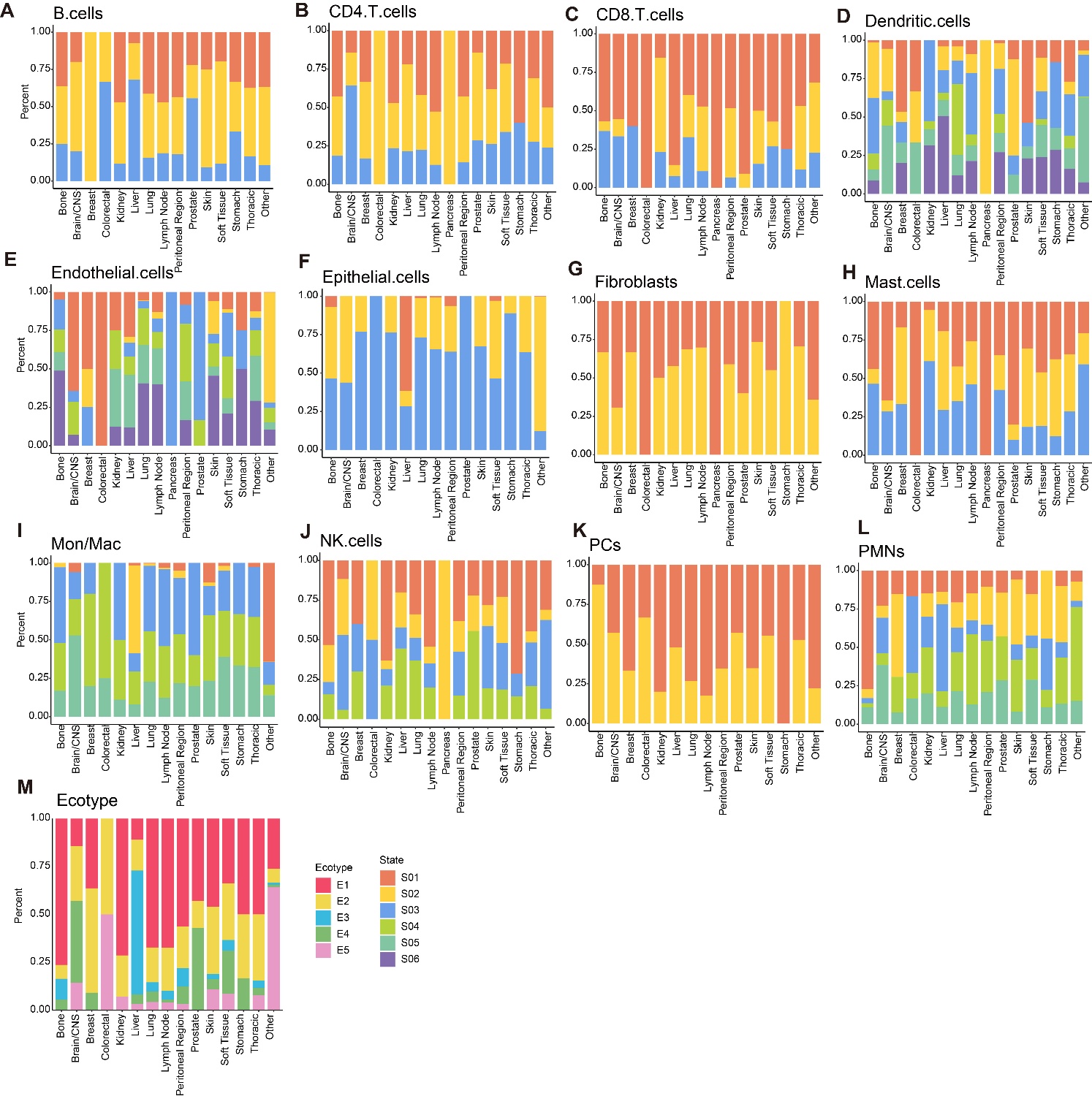


**Supplementary Fig. S11. The landscape of cellular states and ecotypes across different metastatic organs.** Proportions of cellular states and ecotypes across various metastatic organs. **(A)** for B.cells. **(B)** for CD4.T.cells. **(C)** for CD8.T.cells. **(D)** for Dendritic.cells. **(E)** for Endothelial.cells. **(F)** for Epithelial.cells. **(G)** for Fibroblasts. **(H)** for Mast.cells. **(I)** for Mon/Mac. (J) for NK.cells. **(K)** for PCs. **(L)** for PMNs. **(M)** for Ecotype.


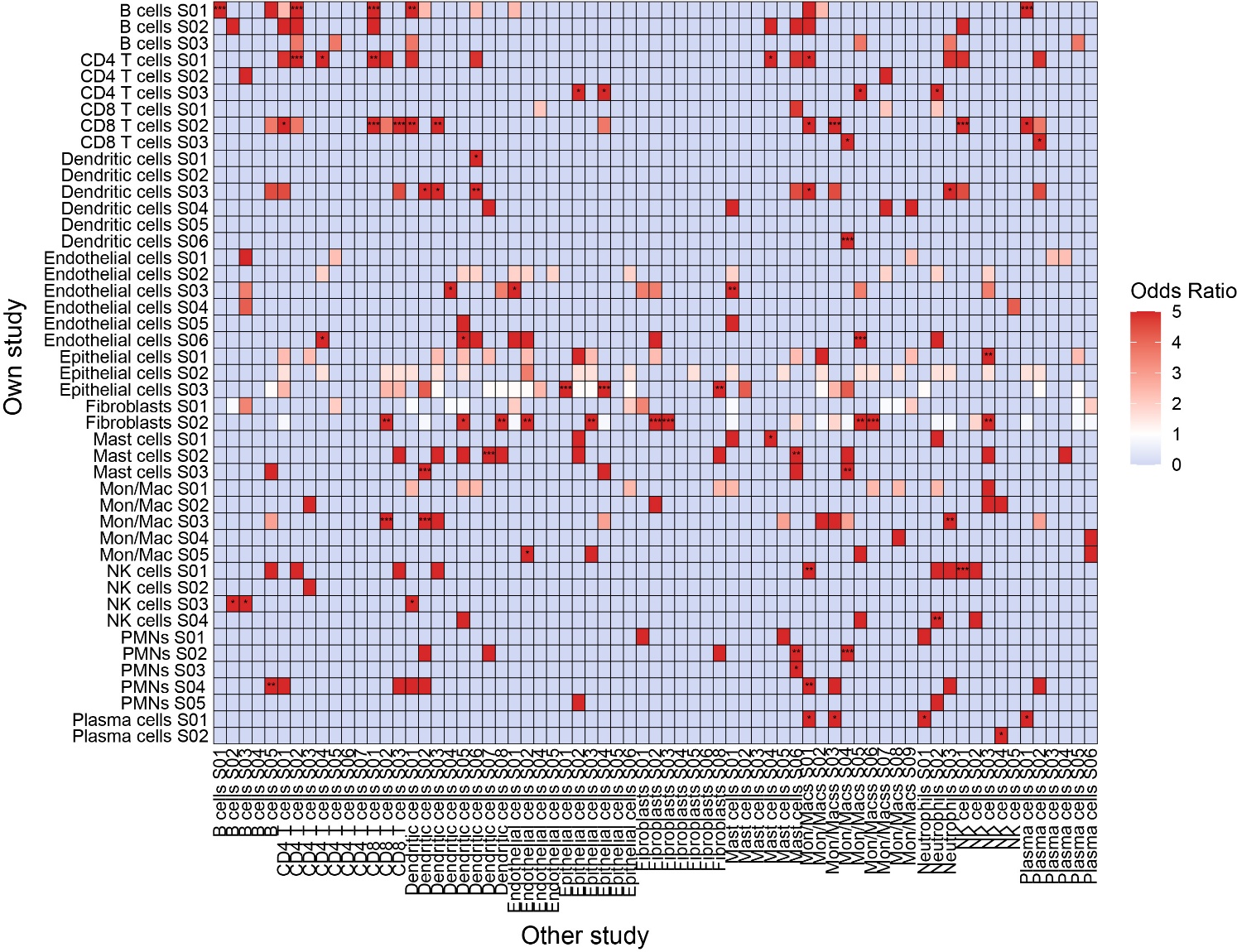


**Supplementary Fig. S12.** Heatmap showing odds ratios assessing for each pair of programs (rows, columns) if they are co-occurrent (≥1, red) or exclusive (<1, blue) than expected by chance (P < 0.05). P values are derived from Fisher’s exact test (* P < 0.05, ** P < 0.01, *** P < 0.001, **** P < 0.0001).


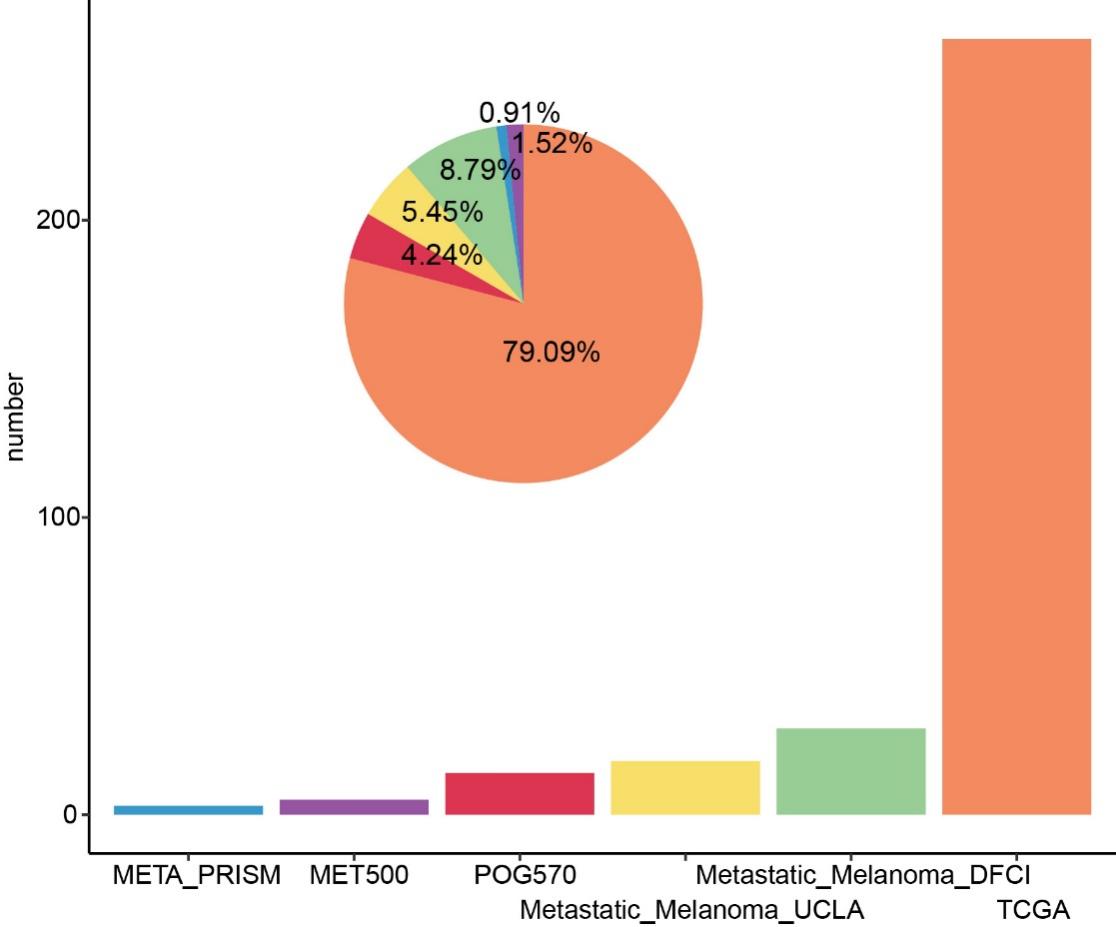


**Supplementary Fig. S13.** The bar chart represented the number of different datasets in E5, and the pie chart represented the proportion of different datasets in E5.


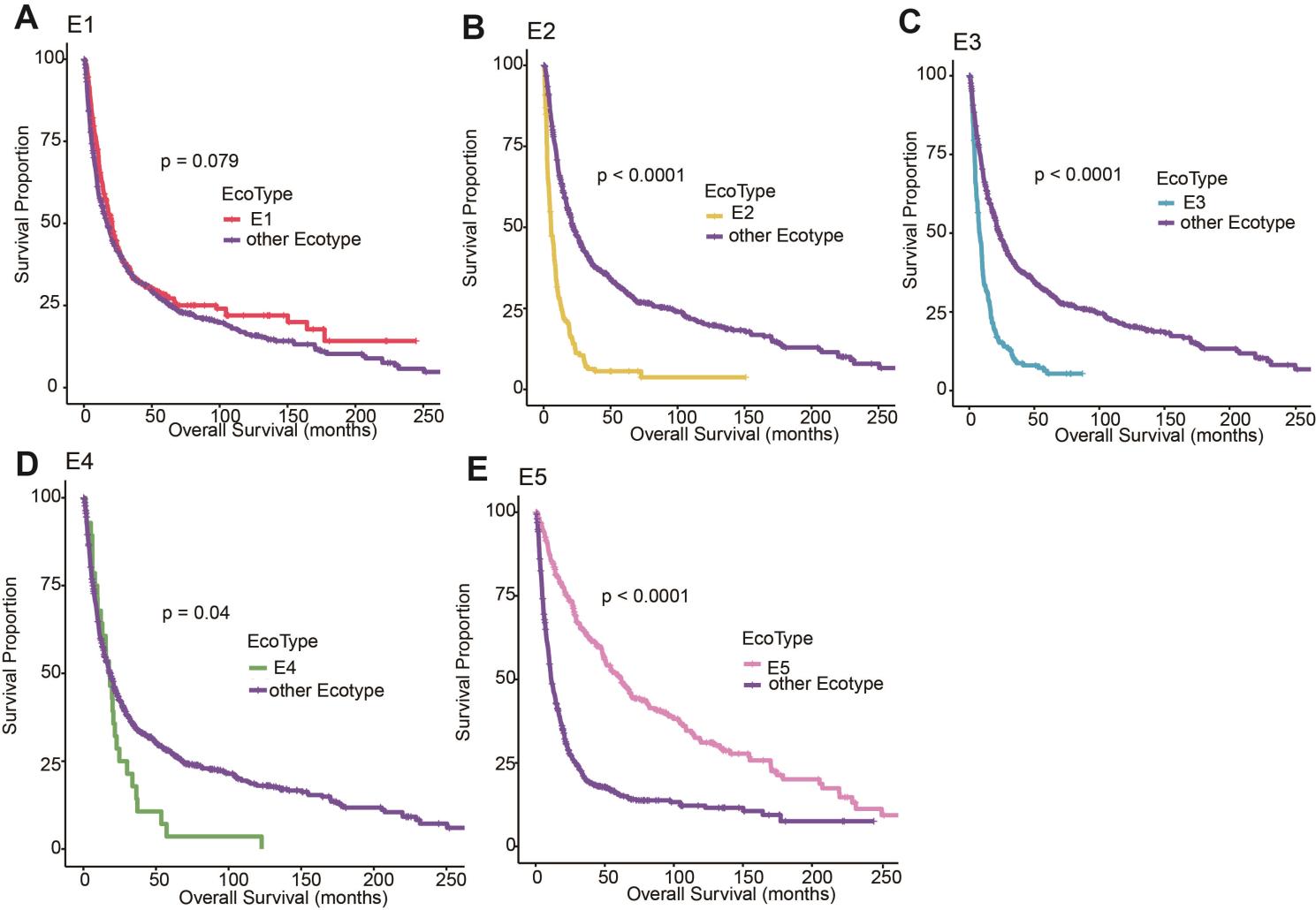


**Supplementary Fig. S14. Ecotypes linked to survival in metastatic cancer patients. (A)** Kaplan–Meier curves showing overall survival differences for patients with E1 and other ecotypes. **(B)** Kaplan–Meier curves showing overall survival differences for patients with E2 and other ecotypes. **(C)** Kaplan–Meier curves showing overall survival differences for patients with E3 and other ecotypes. **(D)** Kaplan–Meier curves showing overall survival differences for patients with E4 and other ecotypes. **(E)** Kaplan–Meier curves showing overall survival differences for patients with E5 and other ecotypes. P-value are calculated using log-rank test.
